# Definition of a cell surface signature for human cardiac progenitor cells after comprehensive comparative transcriptomic and proteomic characterization

**DOI:** 10.1038/s41598-019-39571-x

**Published:** 2019-03-15

**Authors:** José Luis Torán, Juan Antonio López, Patricia Gomes-Alves, Susana Aguilar, Carlos Torroja, Marco Trevisan-Herraz, Isabel Moscoso, Maria João Sebastião, Margarida Serra, Catarina Brito, Francisco Miguel Cruz, Juan Carlos Sepúlveda, José Luis Abad, Carlos Galán-Arriola, Borja Ibanez, Fernando Martínez, María Eugenia Fernández, Francisco Fernández-Aviles, Itziar Palacios, Luis R-Borlado, Jesús Vázquez, Paula M. Alves, Antonio Bernad

**Affiliations:** 1 Department of Immunology and Oncology, Centro Nacional de Biotecnología (CNB-CSIC), Campus Universidad Autónoma de Madrid, 28049 Madrid, Spain; 2 Cardiovascular Development and Repair Department, Spanish National Cardiovascular Research Center (CNIC), Melchor Fernández Almagro 3, 28029 Madrid, Spain; 3 Laboratory of Cardiovascular Proteomics, Spanish National Cardiovascular Research Center (CNIC), Melchor Fernández Almagro 3, 28029 Madrid, Spain; 4 iBET, Instituto de Biologia Experimental e Tecnológica, Apartado 12, 2781-901 Oeiras, Portugal; 5 Instituto de Tecnologia Química e Biológica, Universidade Nova de Lisboa, Av. da República, 2780-157 Oeiras, Portugal; 6 Bioinformatics Unit, Spanish National Cardiovascular Research Center (CNIC), Melchor Fernández Almagro 3, 28029 Madrid, Spain; 7 CIMUS, Avda Barcelona s/n, Santiago de Compostela, 15782A Coruña, Spain; 8 Coretherapix S.L. U. Santiago Grisolia 2, 28769 Tres Cantos, Madrid Spain; 9 Centro Nacional de Investigaciones Cardiovasculares Carlos III (CNIC), Madrid, Spain; 10 Department of Cardiology, Hospital General Universitario Gregorio Marañón, Instituto de Investigación Sanitaria Gregorio Marañón, C/ Dr Esquerdo, 46, 28007 Madrid, Spain

## Abstract

Adult cardiac progenitor/stem cells (CPC/CSC) are multipotent resident populations involved in cardiac homeostasis and heart repair. Assisted by complementary RNAseq analysis, we defined the fraction of the CPC proteome associable with specific functions by comparison with human bone marrow mesenchymal stem cells (MSC), the reference population for cell therapy, and human dermal fibroblasts (HDF), as a distant reference. Label-free proteomic analysis identified 526 proteins expressed differentially in CPC. iTRAQ analysis confirmed differential expression of a substantial proportion of those proteins in CPC relative to MSC, and systems biology analysis defined a clear overrepresentation of several categories related to enhanced angiogenic potential. The CPC plasma membrane compartment comprised 1,595 proteins, including a minimal signature of 167 proteins preferentially or exclusively expressed by CPC. CDH5 (VE-cadherin),  OX2G (OX-2 membrane glycoprotein; CD200), GPR4 (G protein-coupled receptor 4), CACNG7 (calcium voltage-gated channel auxiliary subunit gamma 7) and F11R (F11 receptor; junctional adhesion molecule A; JAM-A; CD321) were selected for validation. Their differential expression was confirmed both in expanded CPC batches and in early stages of isolation, particularly when compared against cardiac fibroblasts. Among them, GPR4 demonstrated the highest discrimination capacity between all cell lineages analyzed.

## Introduction

Adult multipotent cardiac stem cells (CSC) were first defined based on surface expression of the tyrosine kinase receptor c-kit^1^. Other cell surface markers were later proposed to describe resident subpopulations including Sca-1, ATP-binding cassette Abcg2 or PDGFRα. This diversity of potential markers (reviewed in ref. ^2^) has hindered unambiguous identification and molecular definition of endogenous cardiac stem/progenitor cells (CSC/CPC). Similarly, lineage-tracing analyses have yielded somewhat contrasting findings^3–7^.

Murine ckit-CSC were proposed as necessary and sufficient for cardiac regeneration and repair^8^. However, several studies using different strategies for lineage tracing of c-kit+ CSC failed to demonstrated a significant contribution to the cardiomyocyte lineage^9,10^. This controversy prompted a more precise study of c-kit + populations, which concluded that the evident differences seem to be related to the intrinsic limitations of the technique used^11,12^. Current thoughts on these issues are more conciliatory and ckit-expression is considered necessary but not sufficient to define CSC^13^, and the limitations of most lineage-tracing mouse models using c-kit promoter seem evident^11^. It is possible that alternative methodologies such as using pre-characterized BAC constructs^11^ might help to experimentally address this issue.

Evidence from several models is compatible with the involvement of CSC/CPC populations in cardiomyocyte turnover^3,6,14^. An external origin of CSC/CPC is not supported by the evidence, and the focus of the current debate revolves around the direct contribution of mature cardiomyocytes by dedifferentiation/proliferation^4,7^. Low turnover based on resident CSC/CPC is, nonetheless, compatible with a degree of transient dedifferentiation and limited proliferation of pre-existing cardiomyocytes in response to specific signals^15^.

Several lines of evidence from preclinical studies of CSC/CPC transplantation suggest that the observed benefits are due mainly to indirect mechanisms. CSC/CPC protect cardiomyocytes from death and stimulate endogenous repair and regenerative pathways, which lead to long-lasting favorable effects in spite of the short-lived nature of transplanted cells^14,16^. Human c-KIT^+^ CSC/CPC (hereafter denoted CPC for simplification) express *GATA4*, *OTX2*, *SNAI1*, *FOXA2*, *PDX1*, *VEGFR2* and *SOX17* genes^17,18^. In addition, the B7 family protein PD-L1 (programmed death ligand 1) has been shown to be essential for CPC-mediated immunoregulation^18,19^.

The first two clinical trials using cardiosphere-derived cells (CDC) have published their initial phases (CADUCEUS and TICAP), with promising results^20–22^. Both trials confirmed an increase in viable myocardium, resulting in improved regional contractility of the infarcted area, clearly superior to previous findings using any other cell population^23^. However, an integral analysis of CPC/CSC biology and their behavior in response to acute or diffuse chronic damage will be central for a better understanding of the mechanisms involved in these beneficial effects and to improve further treatment strategies.

Based on promising preclinical data^24^, a phase I/IIa clinical trial (CARE-MI; NCT02439398) has been developed using allogeneic expanded CPC populations^25,26^, isolated based on c-KIT expression^17,18^. In an attempt to define the specific protein network associable with expanded CPC, here we have used genomic and proteomic approaches to compare human CPC with human bone marrow mesenchymal stem cells (MSC), a recognized multipotent population, and with human dermal fibroblasts (HDF) as a distant reference population. The results reveal a large group of proteins that are expressed preferentially or specifically in CPC, with a special enrichment of cell surface proteins. These data provide valuable information for further understanding of CPC/CSC activation mechanisms and subsequent cardiac repair processes. Moreover, validated markers could be used in conjunction with c-KIT expression for *ex vivo* or *in vivo* characterization.

## Results and Discussion

### Deep comparative transcriptome analysis of CPC by mRNA sequencing

As a first approach to define specific CPC functions, we used mRNAseq to compare human CPC from three independent donors (CPC1–3) with human bone marrow MSC (*n* = 3; aiming to identify putative genes related to multipotency), and with human dermal fibroblasts (HDF) as a distant reference (*n* = 3; to discard genes expressed similarly in all cell types). CPC were isolated based on cKIT expression as previously described^17,18^, and expanded and studied following the scheme shown in Supplementary Fig. S1. After a preliminary evaluation of the impact of different culture media in CPC *vs*. HDF growth, we  selected to culture each cell type in its optimal medium (Supplementary Methods). CPC were cultured in conditions equivalent to those used for the associated CARE-MI clinical trial^25^; culture medium exchange provoked moderate differences in gene expression, but had no effect on the dominant expression profiles.

**Figure 1:**
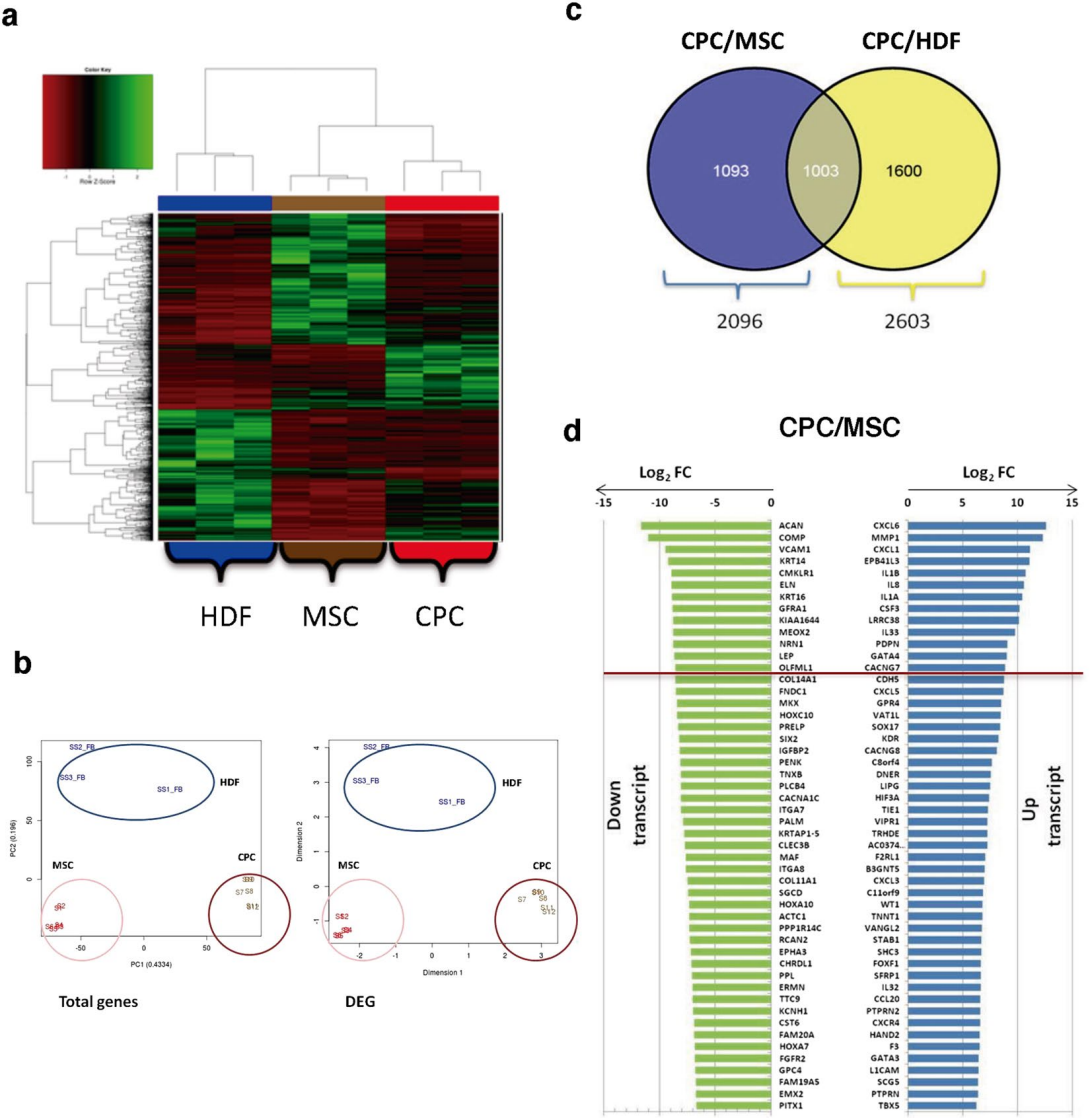
RNAseq analysis of CPC compared with MSC and HDF. (**a**,**b**) mRNAseq experiments were carried out and analyzed using the Ilumina platform, with replicates and/or technical duplicates of all samples (see Methods). Analysis of three CPC isolates (CPC1–3) compared with three MSC (19, 33, 45) and three HDF isolates (F1, F2, F3). Normalized heat map analysis of 12,242 protein-coding genes (**a**) and clustering analysis (**b**) confirmed that CPC, MSC and HDF cell lineages are quite distant and represent significantly differentiated clusters. (**c**) Venn diagram representation of differentially expressed proteins: the specific DEG CPC *vs*. MSC (blue), DEG CPC *vs*. HDF (yellow) and common (grey) genes are represented. (**d**) Plot (log_2_ FC) of top up- or downregulated genes in CPC (CPC1–3) *vs*. MSC (MSC19, MSC33 and MSC45).

CPC, MSC and HDF were compared (FDR < 0.05) only for coding genes from total and differentially expressed gene (DEG) data, using replicates and/or technical duplicates of all samples at the indicated passages (Supplementary Fig. S1). CPC mRNAseq data rendered 12,242 protein-coding genes. Normalized heat map and cluster analysis^27^ confirmed CPC, MSC and HDF as cell lineages significantly different from each other (Fig. 1a,b; see also Supplementary Fig. S2). In addition, we confirmed that the expression profiles were not significantly affected by culture passage, as only 167 out of 11,767 total genes analyzed showed significant variations with passages (Supplementary Fig. S2).

Comparative analysis found 2,096 DEG (17.8%) for the CPC/MSC comparison (p.adj values < 0.05; Fig. 1c), with 1,003 highly preferentially expressed in CPC by simultaneous comparison with HDF (Fig. 1c; Supplementary Table S1). No significant differences were found in association with subcellular compartments (Supplementary Fig. S2). The majority of the top 10 upregulated genes in CPC were specific for the CPC/MSC comparison (Fig. 1d) and were not found upregulated in the CPC/HDF comparison (Supplementary Fig. S2). Among the genes upregulated, C-X-C motif chemokine ligand 6 (*CXCL6*) and matrix metallopeptidase 1 (*MMP1*) showed the maximal differences (Fig. 1d). Other genes of interest not included among the most upregulated genes, such as GATA binding protein 4 (*GATA4)*, calcium voltage-gated channel auxiliary subunit gamma 7 (*CACNG7)*, G protein-coupled receptor 4 *(GPR4) and* cadherin 5 (*CDH5)*, were similarly upregulated in CPC relative to MSC and HDF (Fig. 1d; Supplementary Fig. S2). Aggrecan (*ACAN*) and cartilage oligomeric matrix protein/ thrombospondin-5 (*COMP*) were the more clear examples of down-regulated transcripts in CPC when compared with MSC (Fig. 1d).

### nLC-MS/MS-based comparative proteomic analysis of CPC combined with ITRAQ

**Figure 2:**
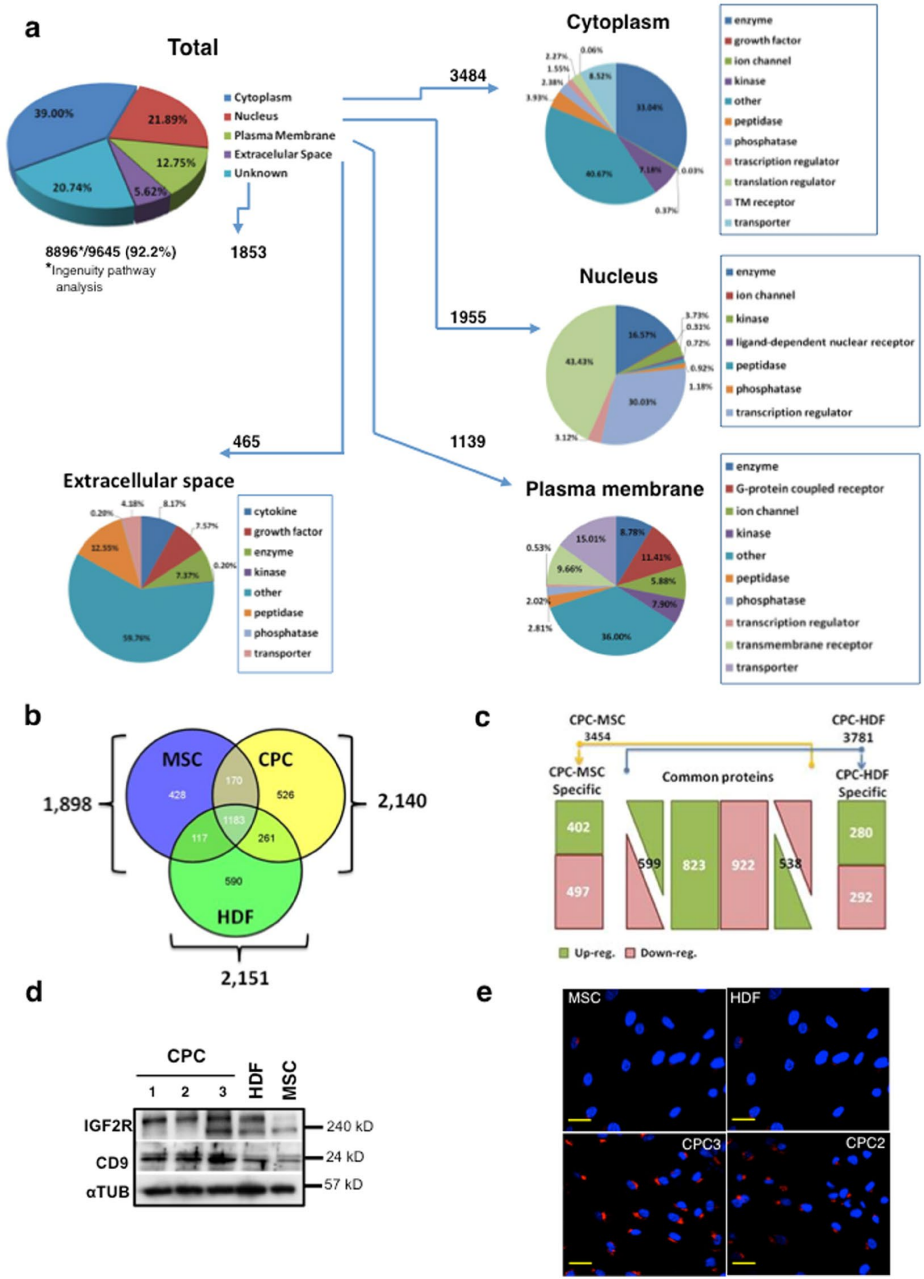
Distribution and classification of CPC proteome compared with MSC and HDF proteomes. (**a**) Distribution of CPC label-free proteomic results, using isolate hCPC3. A total of 9,645 proteins were identified by Uniprot and classified (8,846) by subcellular localization using Ingenuity Analysis Software (IPA); 1,853 proteins were indicated as unknown. For the main subcellular compartments (cytoplasm, nucleus, plasma membrane, extracellular space), circles represent the percentage of protein type function classified by IPA. (**b**) Venn diagram of specific and shared proteins in comparisons of CPC (2,140 proteins), MSC (1,898) and HDF (2,151) proteomes; numbers inside circles indicate specific or shared proteins between CPC, MCS and HDF proteomes. (**c**) Comparison of up- (green) and down-regulated (red) proteins, common to all comparisons (center), specific for CPC in the CPC/MSC (left) or CPC/HDF (right) comparisons, analyzed by  iTRAQ. (**d**) Validation of proteins identified by proteomics. Western blot analysis of IGF2R and CD9 candidate markers in three CPC samples (CPC1–3), HDF (F1) and MSC (MSC19). α-tubulin (αTUB) was used as a loading control; molecular weight (MW; kD) of the proteins analyzed is indicated (right). (**e**) IGF2R immunostaining (red) in two CPC samples (CPC2 & 3), compared with MSC (MSC19) and HDF (F1). Nuclei were DAPI-counterstained. Bars, 20 μm.

The CPC proteome was analyzed by label-free nLC MS/MS (reverse-phase nanoflow liquid chromatography mass spectrometry) in whole cell lysates from the CPC3 isolate. We identified 9,645 proteins (FDR < 0.05), of which 92.2% (8,896 proteins) were classified by Ingenuity Pathway Analysis (IPA). Subcellular protein localization (summarized in Fig. 2a) indicated that 3,484 proteins (39.1%) are cytosolic, 1,955 (21.9%) nuclear, 1,139 (12.7%) plasma membrane and 465 (5.6%) are secreted (Supplementary Table S2). As a second approach to define CPC specific functions, we compared the label-free proteome of CPC1–3 with human MSC (*n* = 3) and HDF (*n* = 3) (FDR < 0.05), and results were classified by IPA. Analysis of CPC *vs*. MSC and CPC *vs*. HDF proteomes showed that 22–29% of the proteins were exclusive to each cell type and 526 (24.6%) proteins were preferentially or exclusively expressed in CPC (Fig. 2b). For a more accurate analysis of differential protein composition, we used ITRAQ (isobaric tags for relative quantitation) (Fig. 2c; Supplementary Fig. S3). Analysis of CPC/MSC and CPC/ HDF proteomes (FDR < 0.05) identified 3,454 and 3,781 proteins, respectively (Fig. 2c); 899 proteins (402 upregulated) were found to be specific for CPC were compared with MSC, and 572 (280 upregulated) when compared with HDF (Fig. 2c). Supplementary Table S3 shows the complete list of differentially-expressed proteins (up- and downregulated) and Supplementary Fig. S3 summarizes the more significant up- and down regulated proteins from the CPC/MSC analysis, organized by differential expression level (Zq). Proteins significantly overexpressed in CPC included insulin-like growth factor 2 mRNA-binding protein 3 (IGF2BP3), interleukin-1 beta (IL1B) and insulin-like growth factor 2 receptor (IGF2R; CD222). As a preliminary validation of data, we analyzed expression of IGF2R. Western blotting showed significantly higher expression in CPC than in MSC (Fig. 2d). Immunofluorescence (Fig. 2e) and FACS (see Supplementary Fig. S4) analyses confirmed a clear IGF2R overexpression. IGF2R, also known as cation-independent mannose-6-phosphate receptor (M6PR), functions in intracellular trafficking of lysosomal enzymes, TGFβ activation and IGF2 degradation^28^; in CPC, IGF2R expression mainly locates at the trans-Golgi network (Fig. 2e).

**Figure 3:**
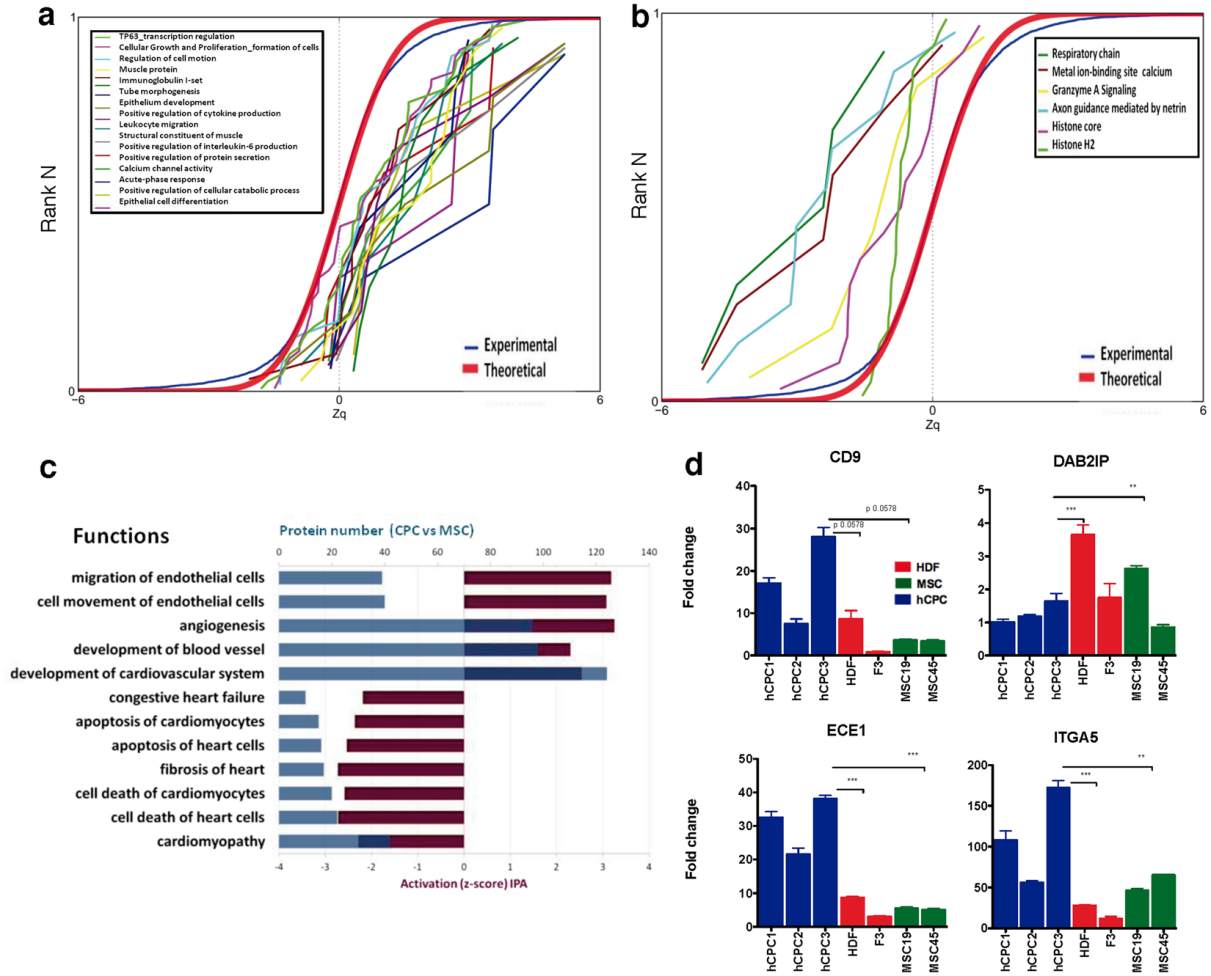
Validation of proteins identified by comparative proteomics and system biology analysis. (**a**,**b**) CPC upregulated (**a**) and downregulated categories (**b**) *vs*. MSC. Proteins represented by three peptides per protein or less (FRD > 5%) were excluded from the analysis. Red line indicates the normal (theoretical) distribution. (**c**) Plot bar from selected protein categories or functions from quantitative CPC/MSC proteomes determine by IPA; for each function, category protein numbers (blue bars, top X axis) and their activation Z-score values (purple bars; bottom X axis) are shown. (**d**) Real-time PCR analysis of *CD9*, *DAB2IP*, *ECE1* and *ITGA5* gene expression in CPC (blue), HDF (red) and MSC samples (green). The assay was performed three times and data are expressed as mean ± SD; black lines indicate the p-value summary (***<0.002, **<0.02, *<0.05) of CPC *vs*. HDF or MSC (one-way analysis of variance followed by the Bonferroni multiple comparison test).

Through systems biology analysis of iTRAQ data, we grouped proteins into functional categories and generated a database (see Supplementary Methods); we assigned a functional category to 85% of the proteins quantified. The results defined several signaling pathways that were up- (Zc+; Fig. 3a) or downregulated (Zc−; Fig. 3b) in CPC in comparison with MSC. Acute phase and positive regulation of cytokine production were significantly overactivated in CPC. Other categories such as muscle protein, Ca^2+^ channel activity and positive regulation of protein secretion were only moderately upregulated in CPC compared with MSC (Fig. 3a).

IPA analysis of the proteins upregulated in the CPC/MSC comparison identified significant differences in discrete categories (Fig. 3c). *Migration of endothelial cells*, *cell movement of endothelial cells* and *angiogenesis* categories had the highest positive scores; also, *development of cardiovascular system*, *blood vessels* and *angiogenesis* categories were well represented (blue bars) among the proteins upregulated in CPC/MSC (122, 97 and 95 proteins, respectively). By contrast, categories such as *congestive heart failure* and *cardiomyopathy* rendered a negative score. From all proteins included in the different *angiogenesis-related* categories (96) a large proportion (58%) were upregulated in the CPC/MSC comparison. To confirm these data, we used RT-qPCR to analyze the differential expression of several examples (Fig. 3d), finding clear overexpression of *CD9* (tetraspanin 29), which was also confirmed by western blotting in CPC compared with MSC and HDF (Fig. 2d). Substantially higher levels of *ECE1* (endothelin-converting enzyme 1) and *ITGA5* (*VLA5A; CD49e*) were also found in CPC. By contrast, *DAB2IP* (DAB2-interacting protein) overexpression was not validated (Fig. 3d). The strong pro-angiogenic activity of CPC compared with MSC has been recently confirmed by comparative secretome analysis^29^, additionally demonstrating an important role for CXCL6, which we also found upregulated by RNAseq (Fig. 1d). These results are in accord with those reported for the CDC used in the CADUCEUS trial^30^, which promote cardiomyocyte proliferation and angiogenesis, and inhibit apoptosis^31^.

### Data integration of the two large-scale techniques

Comparative analysis of mRNAseq (CPC/MSC) with the label-free whole CPC proteome showed 79% cross-identification (7,006 proteins/genes). From the total CPC proteome, 75.5% (6,716 proteins) were also identified by transcriptomics. In addition, comparative RNAseq analysis also defined 1,003 DEG as CPC-specific (Fig. 1d; Supplementary Table S1) and proteomic studies defined 526 CPC-specific proteins, implying 53.5% of cross-identification. A recent deep comparative characterization study of human MSC from different sources using transcriptomics (RNAseq) and quantitative proteomics (nanoLC-MS/MS; SILAC) demonstrated a similar level of overlap, with 60% of data from the proteomics study validated by RNAseq^32^. Important post-transcriptional regulatory mechanisms as well as mRNA ribosome-sorting effects were proposed to explain the degree of overlap, in addition to specific technical limitations and the stringent bioinformatic analysis, which could influence the results on the low-to-medium level-expressed proteins. In our analysis, there was also a fraction of proteins (25%) identified by proteomics that was not reflected in the DEG, which might be explained by the differences in the stability of proteins *vs*. mRNAs^33^. Globally, it has been estimated that ~40% of variation in protein concentration can be explained by mRNA abundance^34^. To explain the remaining ~60% of variation, a combination of post-transcriptional regulation and measurement noise needs to be considered^34^. Therefore, although the expression level of an mRNA might explain only a fraction of the variation in protein abundance, the abundance of a mRNA is often a good proxy for the presence of a protein within the cell.

### CPC surface markers

**Figure 4:**
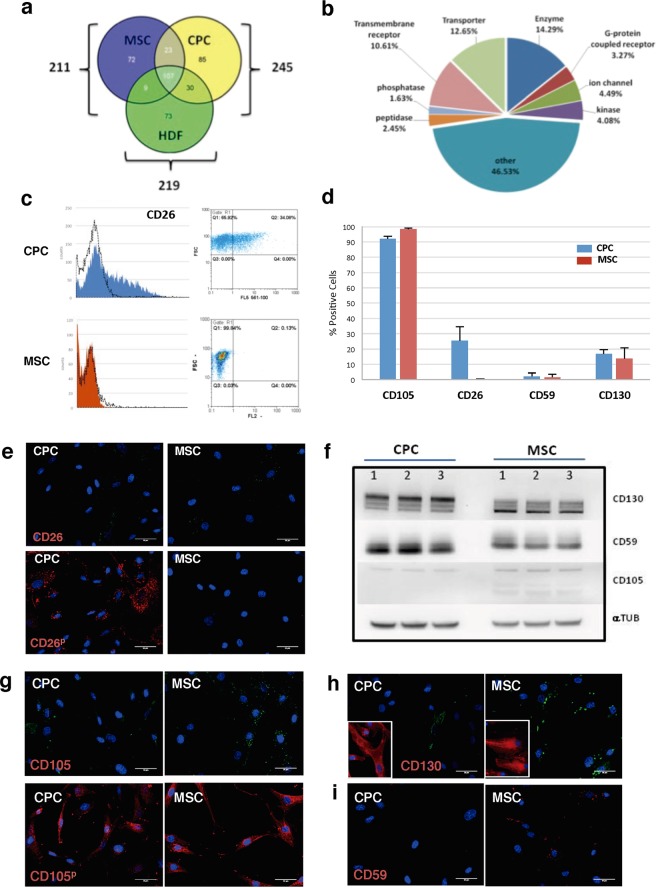
Definition of the minimal core of preferentially expressed plasma membrane proteins in CPC compared with MSC and HDF. (**a**,**b**) Label-free experiments comparing CPC with MSC and HDF; Venn diagram representation of differential upregulated plasma membrane proteins: the specific DEG CPC (yellow), MSC (blue) and HDF (green) genes and common (grey) are represented (**a**); only DEG with p-adjust values < 0.02 were considered. A total of 85 genes were identified exclusively in CPC, with a variety of physiological roles. Relative percentages per specific group of functions, classified by IPA, are indicated (**b**). (**c**,**d**) FACS analysis of CD26 (c), CD105, CD59 and CD130 (**d**) in CPC (blue bars; CPC3) and MSC (red bars; MSC19). The FACS analysis was performed three times using trypsinized cells; data are expressed as mean ± SD. (**e**) Immunofluorescence validation of CD26 in CPC (CPC3) and MSC (MSC19); (^p^) indicates analysis after cell permeabilization; Bars, 20 μm. (**f**) Western blot analysis of CD130, CD59 and CD105 in CPC (CPC1–3) and MSC (*n* = 3); α-tubulin (αTUB) was used as a loading control. After densitometric quantification (upper panel), the CPC/MSC ratio of expression was represented (lower panel). (**g**–**i**) Immunofluorescence validation of CD105 (**g**), CD130 (**h**) and (**i**) CD59 in hCPC (hCPC3) compared with MSC (MSC19); (^p^) indicates analysis after cell permeabilization; insets in (**g**) show cells previously permeabilized. Bars, 20 μm.

To define the CPC membrane-specific or highly preferentially-expressed proteins, we next complemented the deep proteomics strategy with a direct proteomic analysis of enriched membrane fractions. This approach might help to overcome difficulties in receptor identification due to the hydrophobic nature and relatively low abundance of integral membrane proteins. Label-free nLC MS/MS proteomics analysis of CPC yielded 1,139 proteins (11.8% of label-free proteome) classified as plasma membrane proteins according to IPA (Fig. 2; Supplementary Table S4). Comparative analysis of CPC with MSC/HDF identified 85 membrane proteins exclusively expressed in CPC (Fig. 4a; Supplementary Table S5). A previous analysis of enriched membrane fractions from CPC defined a minimum of 1,242 proteins (FDR < 0.05)^28^. Equivalent membrane fractions were obtained from MSC and HDF and analyzed by nanoLC-MS/MS as described^35^. This comparison rendered 27 additional proteins expressed by CPC not previously detected by label-free proteomics of whole extracts. The consolidation of these results revealed a final core of 107 CPC-specific membrane proteins (Fig. 4b). iTRAQ analysis (Supplementary Table S3) confirmed a significant percentage (54%) of these CPC-specific membrane proteins (CPC/MSC comparison), summarized in Table 1. DPP4 (CD26), EPB41L3 and ICAM1 were the most overexpressed membrane proteins in CPC compared with MSC. CD26, which showed the highest overexpression among the membrane proteins in CPC compared with MSC, has been recently linked to regulation of hematopoietic stem/progenitor cells and mature blood cells^36,37^. The most upregulated receptors were TFR1, IGF2R and EPHA2; IGF2R was previously validated (Fig. 2d,e) and some reports propose that CSC secrete IGF2, promoting myocyte differentiation^29,38^.

**Table 1:** Main CPC membrane proteins differentially expressed in comparison with MSC.

Receptome (CPC vs MSC)						
Protein	Description	Alt names	Type-Prot	WP	Mb	ITRAQ (Zq)
DPP4	Dipeptidyl peptidase 4	CD26	Int M Gly	Y	Y	6.81
EPB41L3	Erythrocyte membrane protein band 4.1-like protein 3	DAL1	Int M	Y	Y	4.78
ICAM1	Intercellular adhesion molecule 1	CD54	CS Gly	Y	Y	4.76
TFR1	Transferrin receptor protein 1	CD71	CS-R	Y	Y	3.72
CAP2	Adenylyl cyclase-associated protein 2		Cyt/Nuc	Y	Y	3.36
PLIN2	Perilipin-2		Ves	Y	Y	2.7
IGF2R	IGF Cation-independent mannose-6-phosphate receptor	CD222/M6P-R	Mb-R	Y	Y	3.19
TFR1	Transferrin receptor protein 1	CD71	CS-R	Y	Y	3.72
JUP	Junction plakoglobin	γ-catenin	Mb/Cyt	Y	Y	1.76
CDCP1	CUB domain-containing protein 1	CD318	TM	Y	Y	1.69
EPHA2	Ephrin type-A receptor 2	ARCC2	TK-R	Y	Y	1.67
TNFRSF10B	Tumor necrosis factor receptor superfamily member 10B	CD262/TRAILR2	TM-R	Y	Y	1.61
STIM1	Stromal interaction molecule 1	GOK	TM	Y	Y	1.01
ANK3	ankyrin 3, node of Ranvier (ankyrin G)	Ankyrin G	Cell-Cell	Y	Y	0.87
PLOD1	Procollagen-lysine,2-oxoglutarate 5-dioxygenase 1	PLOD	Enz	Y	Y	0.78
P4HA2	Prolyl 4-hydroxylase subunit alpha		Enz	Y	Y	0.7
LRRC7	Leucine-rich repeat-containing protein 70	LAP1/Densin	TM	Y	Y	0.66
MMP14	Matrix metalloproteinase-14	MT-MMP 1	Enz	Y	Y	0.54
CNTNAP1	Contactin-associated protein 1	Neurexin-4	TM	Y	Y	0.41
VMP1	Vacuole membrane protein 1	TMEM49	Cell-Cell	Y	Y	0.31
PDZD2	PDZ domain-containing protein 2	Papin	Bind to TMR	Y	Y	0.007
HSPG2	Basement membrane-specific heparan sulfate proteoglycan core	PRCAN	Ext-memb	Y	n.id	3.03
NECAP1	Adaptin ear-binding coat-associated protein 1	EIEE21	Clathri-coat. Ves	Y	n.id	2.84
FBLIM1	Filamin-binding LIM protein 1	FBLP-1	Cell junctions	Y	n.id	2.57
SPTAN1	Spectrin alpha chain, non-erythrocytic 1	SPTA2	Scafold prot	Y	n.id	2.11
ITGA3	Integrin alpha-3.	CD49C	Int M	Y	n.id	1.55
EP15R	Epidermal growth factor receptor substrate 15-like 1.		subs	Y	n.id	1.14
TJP2	Tight junction protein ZO-2	ZO2	Tight junctions	Y	n.id	0.76
SLC39A14	Solute Carrier Family 39 (Zinc Transporter), Member 14	ZIP-14	Zn transport	Y	n.id	0.27
CDH5	Cadherin 5, type 2 (vascular endothelium)	CD144	TM	Y	Y	n.id
SEMA4B	Semaphorin 4B	SEMAC	TM	Y	Y	n.id
PPFIA3	Tyrosine phosphatase, receptor type, interacting protein (liprin), alpha 3	LPNA3	Int M	Y	Y	n.id
EFNB1	Ephrin-B1	LERK2	TK-R	Y	Y	n.id
CCDC127	Coiled-coil domain containing 127		Int M	Y	Y	n.id
ABCA2	ATP-binding cassette, sub-family A (ABC1), member 2	ABC2	Transporter	Y	Y	n.id
EMB	Embigin	GP70	Int M Gly	Y	Y	n.id
EPHA4	EPH receptor A4	TYRO1	TK-R	Y	Y	n.id
FADS1	fatty acid desaturase 1	FADSD5	Enz	Y	Y	n.id
IL1R1	interleukin 1 receptor, type I	IL-1R-Alpha	TK-R	Y	Y	n.id
KCNT1	Potassium Channel, Sodium Activated Subfamily T, Member 1	KCa4.1	K channel	Y	Y	n.id
MPP5	Membrane protein, palmitoylated 5 (MAGUK p55 subfamily member 5)	PALS1	Int M	Y	Y	n.id
VLDLR	Very low density lipoprotein receptor	CAMRQ1	Int M-Endo	Y	Y	n.id
TTYH3	Protein tweety homolog 3		Cl^-^ channel	Y	Y	n.id
SLC7A1	High affinity cationic amino acid transporter 1	CAT1	aa-channel	Y	Y	n.id
TNFRSF10D	Tumor necrosis factor receptor superfamily member 10D	CD264/TRAILR4	TM-R	Y	Y	n.id
F11R	Junctional adhesion molecule A	CD321/JAMA1	Int M	Y	Y	n.id
TRPM4	Transient receptor potential cation channel, subfamily M, 4	LTrpC4	Cation channel	n.id	Y	2.06
CCDC47	Coiled-coil domain-containing protein 47	MSTP041	TM	n.id	Y	0.33
EGFR	Epidermal growth factor receptor	HER1	TK-R	n.id	Y	n.id
ACVR2A	Activin A receptor, type IIA	ACVR2	STK-R	n.id	Y	n.id
GPR98	G protein-coupled receptor 98		GPCR	n.id	Y	−0.12
ITGA2	Integrin, alpha 2	CD49B	Subunit coll-R	n.id	Y	−1.31
CD276	CD276 antigen	B7-H3	TM- regulator	n.id	Y	−1.68
CD59	CD59 glycoprotein. MAC-inhibitory protein	MAC-IP	CS Gly	n.id	Y	−2.59

Table summarizes the main examples of differentially (Zq) expressed membrane proteins in CPC compared with MSC by iTRAQ analysis (Supplementary Table S3). Proteins are grouped according to the criteria that they were also found differentially expressed by label-free proteomics of whole extracts (WP) or purified membane fractions (Mb); (n.id; non identified). The last group of proteins are examples for the 27 proteins found only with purified membane fractions; only part of them were validated by ITRAQ.

mRNAseq + IPA analyses defined 342 membrane-associated DEG in CPC (Supplementary Fig. S5), compared with MSC (153) and HDF (189); these included 107 transmembrane receptors and 139 G protein-coupled receptors (GPCR) (Supplementary Fig. S5). The comparative RNAseq analysis yielded a minimal core of 85 plasma membrane proteins that were specifically overexpressed in CPC compared with MSC/HDF. These included nine GPCR (e.g., *CXCR4*, *GPR4*, *VIPR*, *FZD8*) and eleven transmembrane receptors (among them *CD93*, *CD274* and *CD200*) (Supplementary Fig. S5). Expression of the co-stimulatory molecule CD274 (PD-L1) was previously demonstrated, which endows CPC with the capacity to drive significant allogeneic Treg responses and to attenuate ongoing immune response^18^. Our results confirmed *CD200* (*OX2G*) overexpression, which is also involved in immunoregulation and tolerance^39,40^ and in MSC-related bone physiology^41^.

To validate some of these results, we tested the inferred differential expression of dipeptidyl peptidase 4 (DPP4; CD26), CD59, endoglin (CD105) and CD130 proteins in CPC *vs*. MSC by FACS analysis. Results showed clear CD26 overexpression in CPC (18–32%) compared with a relatively low expression in MSC (Fig. 4c,d). Also, CD26 immunofluorescence analysis confirmed its overexpression in CPC compared with MSC (Fig. 4e), which was more evident with permeabilized cells (Fig. 4e, bottom panels). FACS analysis showed that endoglin (CD105), CD130 and CD59 were expressed at similar levels in CPC and MSC (Fig. 4d). Western blotting (Fig. 4f) confirmed moderate upregulation of CD130 (2.5-fold) and CD59 (4.25-fold), but a similar expression of endoglin (CD105). Immunofluorescence analysis revealed the similar expression of CD105 and CD130 (Fig. 4g,h) and a moderate increase in CD59 expression in MSC (Fig. 4i). These results serve to illustrate the complexity of the validation experiments, which is likely related to the previously discussed strong levels of post-transcriptional regulation. Nonetheless, these analyses globally validated IGF2R (CD222) and DPP4 (CD26) as membrane proteins that are significantly overexpressed in CPC in comparison with MSC/HDF.

The minimal RNAseq-based DEG profile (Supplementary Fig. S5) correlated only partially (25%) with the 107 proteins defined by proteomics (Fig. 4a,b; Supplementary Fig. S6; Table S5). Therefore, we focused on this subgroup of 20 plasma membrane-associated proteins verified to be overexpressed in CPC *vs*. MSC/HDF, both by mRNA expression and proteomics analysis (see Supplementary Fig. S6).

**Figure 5:**
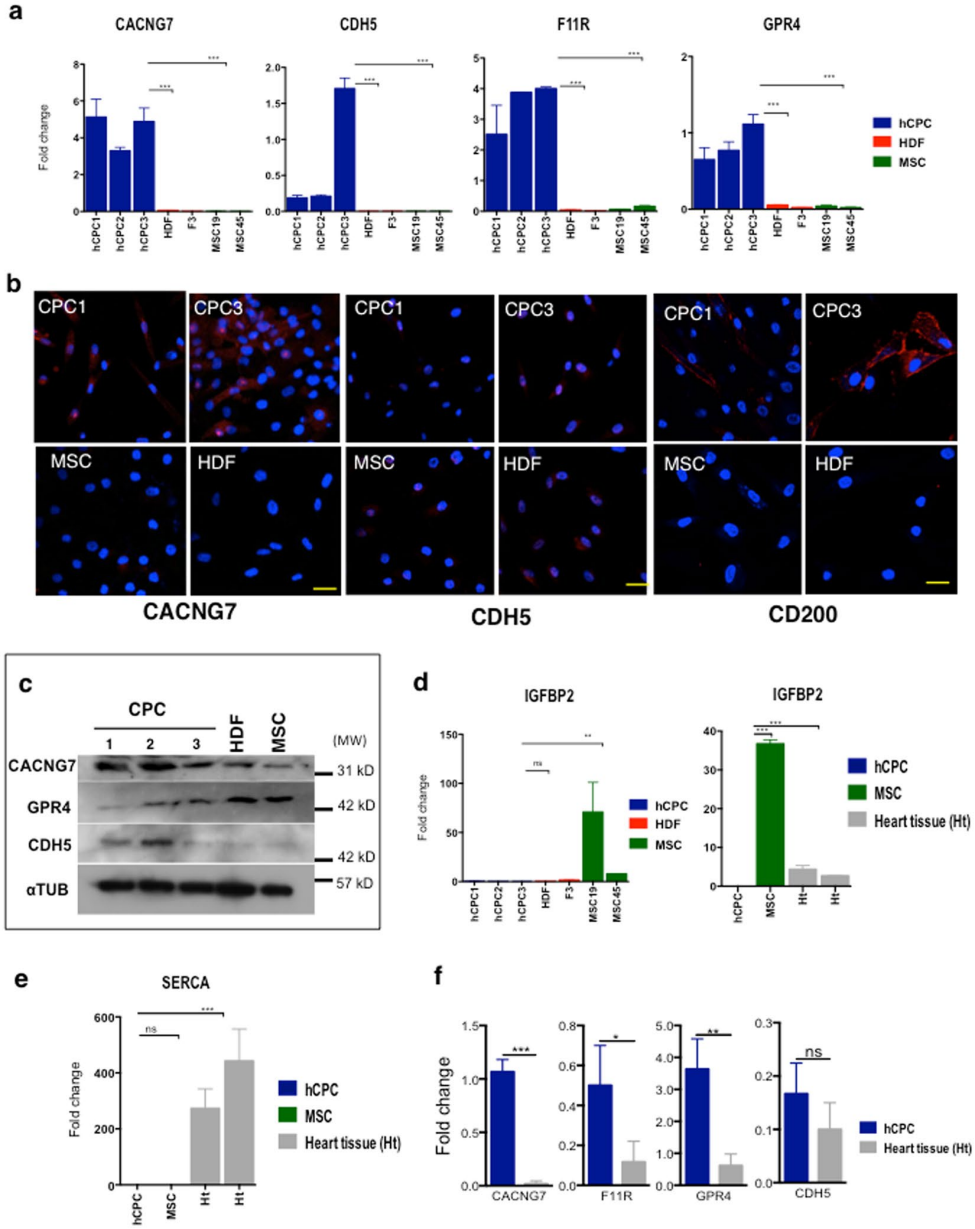
Validation of putative CPC membrane markers. (**a**) RT-qPCR of *CACNG7*, *CDH5*, *FR11*, *GPR4* gene expression from independent CPC donors (CPC1–3; blue bars), two HDF (HDF and F3; red bars), and  two independent MSC (MSC19, MSC45; green bars). (**b**) Immunofluorescence validation of CDH5, CACNG7 and CD200 in CPC samples (CPC1 & 3), compared with MSC (MSC19) or HDF (F1), as controls. Bars, 20 μm. (**c**) Western blot analysis of CACNG7, GPR4 and CDH5 markers was performed in three CPC isolates (CPC1–3), with MSC (MSC19) and HDF as controls. α-tubulin (αTUB) was used as a loading control; molecular weight markers (MW; kD) are indicated (right). (**d**,**e**) RT-qPCR of *IGFBP2* (**d**) and *SERCA* (**e**) gene expression from independent CPC donors (CPC1–3; blue bars), two HDF (HDF and F3; red bars), two independent MSC (MSC19, MSC45; green bars) and two independent human heart samples (grey bars). (**f**) Relative expression (RT-qPCR) of *CACNG7*, *F11R*, *GPR4* and *CDH5* in CPC (blue bars) compared with total human heart tissue (grey bars); values relative to *GAPDH* expression. The assays were performed three times and data expressed as mean ± SD; black lines indicate the p-value summary (***<0.002, **<0.02 *<0.05, ns = not significant) of CPC *vs*. HDF, MSC or heart tissue (one-way analysis of variance followed by the Bonferroni multiple comparison test).

Significant upregulation (p < 0.001) of *CACNG7* and *CDH5* expression was confirmed by RT-qPCR using three CPC isolates as compared with MSC and HDF, where expression was negligible (Fig. 5a). Preferential expression in CPC was also confirmed by western blotting (Fig. 5c). Additionally, differential expression of CACNG7 was confirmed by immunofluorescence analysis (Fig. 5b). Another protein, F11R (JAM-A; CD321), was also clearly confirmed overexpressed by RT-qPCR in CPC *vs*. MSC/HDF (Fig. 5a). Finally, RT-qPCR (Fig. 5a), but not western blottting (Fig. 5c), confirmed high levels of *GPR4* in CPC compared with MSC/HDF (Fig. 5a), suggesting important post-transcriptional regulation.

**Figure 6:**
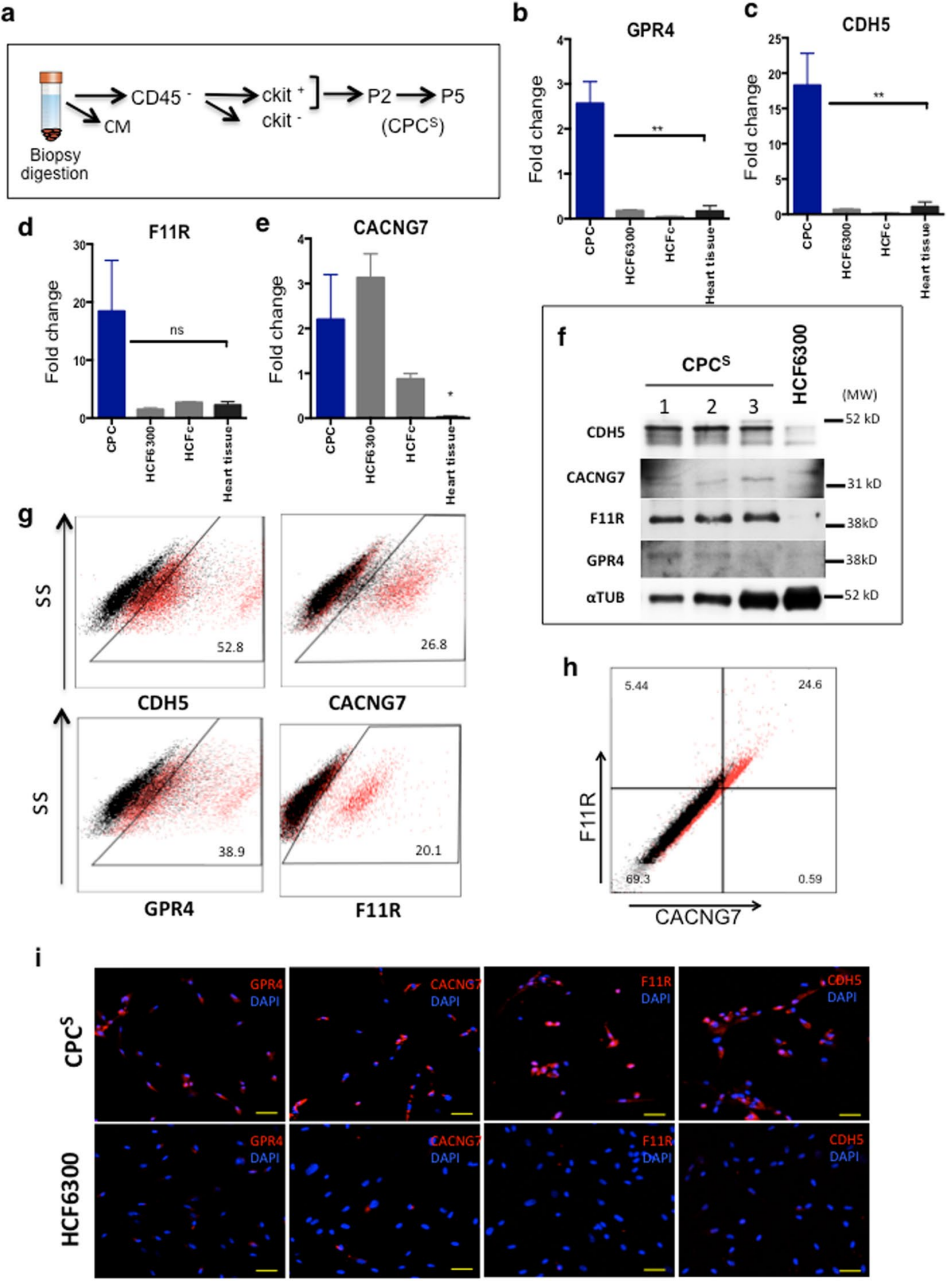
Validation of highly differentially expressed membrane proteins in CPC at early passages after isolation. (**a**) Scheme for the isolation and expansion of human and porcine CPC. Cells were analyzed in passage 2 (p2) or passage 5 (p5); CM cardiomyocytes (**b**–**e**). Relative expression (RT-qPCR) of *GPR4* (**b**), *CDH5* (**c**), *F11R* (d), *CACNG7* (**e**) in CPC^S^ (p2) (blue bars) compared with total human heart tissue (black) bars and cardiac fibroblasts (HCF6300, HFCc; grey bars); values relative to *GAPDH* expression. The assays were performed three times and data expressed as mean ± SD; black lines indicate the p-value summary (***<0.002, **<0.02 *<0.05, ns = not significant); one-way analysis of variance followed by the Bonferroni multiple comparison test). (**f**) Western blot analysis of CACNG7, GPR4, F11R and CDH5 markers was performed in three CPC isolates (CPC1–3), compared with the cardiac fibroblast HCF6300. α-tubulin (αTUB) was used as a loading control; molecular weight markers (MW; kD) are indicated (right). (**g**,**h**) FACS analysis for the indicated simple markers (**g**) and the CACNG7/F11R double labeling (**h**). (**i**) Immunofluorescence validation of GPR4, CACNG7, F11R and CDH4 in a CPC sample (CPC1), compared with the cardiac fibroblast HCF6300 line. Nuclei were stained with DAPI. Bars, 50 μm.

To validate downregulated functions, we used RT-qPCR to analyze *IGFBP2* (*IBP2*; insulin-like growth factor binding protein 2) expression in CPC and MSC. The *IGFBP2* profile was almost specific for MSC (Fig. 5d). All genes found preferentially regulated in CPC and validated (only the expression profile for *IGFBP2* is shown; see Fig. 6) were also confirmed in comparison with several human heart samples; *SERCA*2 was used as cardiac positive control (Fig. 5e).

Finally, the putatively specific CPC plasma membrane repertoire was then challenged with the expression profiles previously described for other proposed cardiac stem/progenitor populations such as cardiosphere-derived cells (CDC) and ckit+ CSC, both from mouse and human origin. In addition, we compared our results with the Sca1 + CSC and the novel murine CPC population, characterized by high expression of BMi1 (B-CPC)^15,42^. Supplementary Fig. S7 shows a representative summary of plasma membrane genes/proteins whose expression is highly conserved among all compared populations (dark green); in addition, the figure also includes some examples of genes that show significant differences among the compared populations (e.g., *CD34*, *CD40* and *CD133*). Although some expression data were not available for all compared populations, in conclusion, the human CPC surface membrane expression profile defined here is compatible with published data from both human CDC and ckit-CSC, albeit with some differences including expression of *ICAM1*, *ICAM2*, *PEPN*, *PDGFRA*, *PROM1*, *CD40*, *CD13* and *Sema-7A*, between human CPC and human CDC (Supplementary Fig. S7).

### Validation of markers for human CPC

Based on the sizeable differences in the levels of overexpression in CPC *vs*. MSC/HDF and previous successful pre-validations (Fig. 5), *CDH5 (VE-cadherin)*, *GPR4*, *CACNG7*, *F11R (JAM-A; CD321) and CD200 (OX2G)* were selected for validation. *CDH5* and *CD200* are clearly overexpressed in CPC *vs*. MSC, and are similarly expressed by all CPC/CSC populations reported in the literature (Supplementary Fig. S7). Concerning *F11R and CACNG7*, although less data are available they are compatible, with our results, demonstrating a clear but lower ratio in CPC/MSC. Finally, *Cdh5*, *Cd200 and F11r* were also confirmed overexpressed in the more immature murine B-CPC population in comparison with the reference population^42^, and in ckit^+^ CSC^43^ (Supplementary Fig. S7).

The expression levels of all putative surface markers for human expanded CPC were compared with whole human cardiac tissue by RT-qPCR. In contrast to *CACNG7*, *GPR4* and *F11R*, which were preferentially expressed by CPC, *CDH5* overexpression was lower and not statistically significant (Fig. 5f). As a final validation on expanded CPC, we compared the four putative positive markers for CPC and a negative marker (IGFBP2) in three independent isolates (CPC1-3) against cardiac fibroblasts (HCF6300 and HCFc), fibroblast from other origins (HDF and F3) and bone marrow MSC (MSC19, MSC 45). *GPR4* demonstrated a robust preferential expression in CPC and *IGFBP2* was clearly not expressed in CPC compared with the remainder of cells tested (Supplementary Fig. S8). Preferential expression of *CACNG7* was also statistically significant (Supplementary Fig. S8). By contrast, *F11R* expression, although clearly preferentially expressed in CPC, was not statistically significant in comparison with cardiac fibroblasts (Supplementary Fig. S8). Finally, discrimination against cardiac fibroblasts by *CDH5* expression was poor (Supplementary Fig. S8). Overall, these results confirm the potential use of *GPR4* and *CACNG7* as useful positive markers (and *IGFBP2* as a negative marker) for the characterization of expanded CPC.

To test the robustness of these markers, and to discard the possibility that their expression profile was significantly associated with the culture expansion conditions, we sought to confirm the expression of this small panel for CPC in early stages (p2–p5) of isolation/expansion (Fig. 6a). We named these populations CPC^S^ (for short-term expanded CPC), to differentiate them from expanded CPC. RT-qPCR analyses confirmed a statistically-significant overexpression of *GRP4* (Fig. 6b) and *CDH5* (Fig. 6c) as compared with cardiac fibroblasts (HCF6300 and HCFc) and heart tissue. *F11R* was also demonstrated to be overexpressed but differences were not statistically significant (Fig. 6d). Conversely, expression of *CACNG7*, although overexpressed with respect to heart tissue (Fig. 6e), did not show clear differences with the level of expression in cardiac fibroblasts. Western blotting confirmed results with the exception of CACNG7 (Fig. 6f).

In addition, we analyzed CPC^S^ by FACS (Fig. 6g). In comparison with their corresponding isotype controls, CDH5 showed (at p2) the greatest expression (52.8%) in CPC^S^, followed by GPR4 (38.9%), CACNG7 (26.8%) and F11R (20.1%). As a possibly interesting combination of expressed markers, based also on their functions in other stem cell systems^44–46^, we analyzed co-expression of F11R and CACNG7 in CPC^S^ in early stages (p2) by FACS. The results of the analysis showed heterogeneity in the population, revealing that about 25% of the primary CPC^S^ were double-positive cells (Fig. 6h), and suggesting that the majority of CACNG7^+^ cells are also F11R^+^.

Immunofluorescence analysis confirmed the clear overexpression of the four markers, including also CACNG7, in human CPC in early stages (p2–p5), compared with cardiac fibroblast HCF6300 cells (Fig. 6i). The strong variation observed between mRNA and protein for CACNG7 is likely related to post-transcriptional regulation.

Overall, our data show that GPR4, CDH5 and F11R fulfill all the criteria to be highly preferentially expressed in CPC compared with the other cell lineages analyzed, and in particular with cardiac fibroblasts. In addition, we have demonstrated that they are all expressed at high levels soon after isolation. Because the global comparative analysis (genomics vs proteomics) has been performed using expanded populations, it is important to remark that some of the genes/proteins identified as preferentially expressed in CPC could be modulate by *ex vivo* expansion. This must be evaluated for each individual gene/protein.

As a preliminary evaluation of the potential conservation of these putative markers for CPC, expression of *F11R and CACNG7* was evaluated in 2 independent isolates of porcine CPC (pCPC) and compared with human CPC. Results demonstrated that both genes were similarly expressed (Supplementary Fig. S8). Analysis at the early stages of pCPC isolation (p2–p5) also demonstrated a clear overexpression of *pCACNG7* compared with whole heart tissue, whereas the *pF11R* expression pattern was not as evident. (Supplementary Fig. S8). Finally, due to the limited cross-reactivity of the available antibodies (human/pig), we could only evaluate pCDH5 expression in early (p2) passages by FACS. Similar to the results in human CPC, 62% of pCPC (p2) cells expressed significant levels of pCDH5 although with less intensity than in long-term expanded pCPC (Supplementary Fig. S8).

These results confirm that the combined expression of GPR4, CACNG7, F11R and CDH5 defines a heterogeneous population of human CPC, isolated based on c-KIT+ expression and expanded in the conditions equivalent to that used in the CARE-MI clinical trial^25,26^. All markers are expressed in c-KIT+ CPC soon after isolation, and mostly maintained, both in human and pig cells during *ex vivo* expansion. Taking all this evidence together, GPR4, F11R, CACNG7 and CDH5 are human CPC surface-expressed proteins that can be used in combination with c-KIT, for a variety of downstream applications.

CDH5 plays a critical role in endothelial adherence junction assembly and maintenance, through homophylic interactions, and contributes to flow sensing by endothelial cells. In addition, the CDH5 transmembrane domain has been shown to interact with transmembrane domains of VEGFR2, as well as VEGFR3, forming part of the junctional mechanosensory complex to facilitate ligand-independent transactivation^44^. It is therefore tempting to speculate that CDH5 could play a similar role in CPC, participating in the regulation of CPC activity *via* mechanosensory imputs, although more work is needed to test this hypothesis. F11R was also clearly confirmed as overexpressed in CPC *vs*. MSC/HDF, demonstrating a substantial overexpression with respect to cardiac fibroblasts (Fig. 6d). F11R is essential for regulating Notch signaling in hematopoietic stem cells as well as in mesoangioblast extravasation^45^, and F11R blocking antibodies greatly enhance mesoangioblast engraftment in dystrophic muscle^46^. A similar role could be envisioned in CPC.

More intriguing is the potential role of CACNG7 and GPR4, which are significantly and preferentially expressed (particularly at the protein level for CACNG7) in CPC. CACNG7 (also known as *TARP γ-7*) is the voltage-dependent calcium channel gamma-7 subunit, acting also as a regulatory protein (trafficking and gating) for transmembrane AMPA receptors. Although initially defined as specific for the brain, it was later confirmed to be expressed by atrial and ventricular myocytes, and to be downregulated by cardiac ischemia. Also, it has been demonstrated that CACNG7 transcriptionally regulates Ca(V)2.2 channels, down-regulating N-type currents^47,48^. Finally, CACNG7 is preferentially downregulated in brain tumor stem cell types as compared with normal neural stem cells, and is also downregulated in several other cancer models^49^. Taken together, we can speculate that CACNG7 expression could form part of a specific program in CPC to favor their immature state by Ca^2+^ signaling control. Indeed, a strong correlation between low Ca^2+^ signaling and quiescence has been recently demonstrated both in hematopoietic stem cells^50^ and glioblastoma stem-like cells (GSLC)^51^. These interesting observations will be addressed in future research.

Finally, GPR4 is a proton-sensing GPCR that might also sense amino acids, pointing to its role in many intracellular signaling pathways^52^. Acidosis commonly exists in the tissue micro-environment of various pathophysiological conditions such as tumorigeneisis, inflammation, ischemia, metabolic disease, and respiratory disease. However, how the acidic microenvironment affects the function of blood vessels is not yet well defined. GPR4 is expressed by endothelial cells and plays an important role in mediating ER stress response induced by acidosis^53^, coordinated through the Notch pathway^54^. Alterations in extracellular pH also affect quiescence of stem cells, as lowering of pH favors quiescence of GSLC through the remodeling of Ca^2+^ signaling^51^. However, no specific role for GPR4 has been reported to dat in any stem cell model.

## Conclusions

Using a combination of RNA sequencing and quantitative MS-based proteomics, we report here the most comprehensive proteome to date for human adult cardiac c-KIT+ progenitor cells, compared with human MSC and HDF. Both techniques demonstrate high similarity of expression profiles; 75.5% of the CPC proteome was represented in the transcriptome data. RNA sequencing allowed the identification of 1,003 DEG when compared with MSC and HDF, while MS-based proteomics yielded 526 DEG proteins. Systems biology analysis of quantitative proteomics showed a clear overrepresentation in CPC of categories associated with angiogenic potential.

A minimal combined specific CPC plasma membrane signature consisting of 167 genes has been defined. Among the CPC core functions that were confirmed both by genomics and proteomics *CDH5*, *GPR4*, *CACNG7*, *CD200* and *F11R* were validated in human and porcine samples. GPR4 is the CPC marker that showed the best discrimination capacity against all cell lineages analyzed as well as against human cardiac tissue.

## Methods

### Cell culture

Human bone marrow-derived MSC and human dermal fibroblasts were obtained from the Inbiobank Stem Cell Bank (www.inbiobank.org). Briefly, cadaver bone marrow was harvested from brain-dead donors, under consent, with the supervision of the Spanish National Transplant Organization (*Organización Nacional de Trasplantes*, ONT). Passages of the different cultures used for the different studies are indicated specifically in the corresponding figures or legends to figures. Human CPC were obtained from right atria appendage, after positive evaluation by the Ethical and Research Committee of Hospital General Universitario Gregorio Marañón (HGUGM). Porcine CPC were obtained from the cardiac appendage, after positive evaluation by the Ethical and Research Committee of the National Center for Cardiovascular Research (CNIC). Research was carried out according to The Code of Ethics of the World Medical Association (Declaration of Helsinki). Primary human and porcine CPC isolates were obtained as previously indicated^12^ (Supplementary Information) and maintained for the indicated passages.

### Label-free proteomics analysis

Cells from CPC isolates hCPC1-3 were used. A working cell bank was prepared (from P4 and P5, for hCPC2 and hCPC1&3, respectively) and they were expanded up to P7 and P8, respectively. After several washes in PBS, cell pellets (5–8 × 10^7^) were collected and aliquoted. For the deep proteomic analysis, isolate hCPC3 was exclusively used, using biological triplicates. For protein extract preparation, pellets were resuspended in lysis buffer (50 mM Tris-HCl pH 8.5, 4% sodium dodecyl sulphate (SDS) and 50 mM dithiothreitol, boiled (5 min) and incubated (30 min, room temperature) for full protein solubilization. Total protein (~200 mg) was processed (see Supplementary Methods), the resulting tryptic peptides dissolved in 0.1% formic acid and loaded into the nLC-MS/MS system. To increase proteome coverage, tryptic peptides were fractionated by cation exchange chromatography (Oasis HLB-MCX columns), which were desalted and analyzed using reported system and conditions (see Supplementary Methods). Peptide identification and quantification is described in Supplementary Methods.

### iTRAQ labeling and quantitative proteomics

Equal amounts of digested peptide samples were labeled with the 4-plex iTRAQ (isobaric tags for relative and absolute quantitation) Reagents Multiplex Kit (Sciex); reactions were terminated by incubating samples with 0.5% (v/v) trifluoroacetic acid. Labeled peptides were mixed, concentrated in a SpeedVac, desalted in C18 Oasis-HLB cartridges and dried for mass spectrometry analysis. iTRAQ-labeled peptides were analyzed on a Q Exactive Hybrid Quadrupole-Orbitrap mass spectrometer (Thermo Scientific) using conditions as reported (see Supplementary Methods). Peptides were identified and quantified as described in Supplementary Methods.

### Systems biology

For systems biology analysis, proteins were grouped into functional categories from a database created by joining categories and pathways from Gene Ontology, Reactome, PIR, and KEGG Pathways (all retrieved using the DAVID bioinformatics resource^54,55^), as well as Ingenuity Pathway Analysis databases (www.ingenuity.com; versions 12 August 2014). This classification included a total of 16,763 proteins, 5239 of which were among the 6108 proteins quantified; 85% of quantified proteins were thus indicated in at least one category. In total, 14,573 categories were present in the database, for a total of 713,289 protein-category relations. As for spectra, peptides and proteins, we calculated an averaged log_2_ ratio at the protein category level, ${X}_{c}$, as well as the corresponding normalized value ${Z}_{c}$, to detect the categories containing proteins significantly over- or under-expressed. Using this approach, only categories with at least five proteins were considered.

### Flow cytometry

CPC, MSC or HDF were detached with trypsin-EDTA and washed with PBS and 0.01% bovine serum albumin (BSA). Cells were incubated with primary antibodies or isotype controls (1 h, on ice) (Supplementary Methods). After extensive washing, cells were incubated with fluorescent secondary antibody (30 min, on ice), washed with PBS + 0.01% BSA and analyzed on a FACS Canto 3 L flow cytometer (BD Biosciences).

### Western blotting

Cells were harvested in RIPA (radioimmunoprecipitation assay) lysis buffer and equal amounts of lysates were separated by 10% SDS-PAGE. Proteins were transferred to PVDF membranes using the iBlot Dry Blotting System (Invitrogen). After incubation with primary and secondary antibodies, signals were developed using an ECL kit (GE Healthcare).

### Immunofluorescence

Antibodies and dilutions used are summarized in Supplementary Methods. Cells were fixed in 4% paraformaldehyde (PFA), blocked with blocking buffer (PBS with 10% fetal bovine serum, FBS; 30 min, room temperature), permeabilized (5 min, room temperature) with 0.1% Triton-X100, and incubated with primary antibodies (overnight, 4 °C). After washing, cells were incubated with an appropriate secondary antibody (1 h, room temperature); washed cells were mounted in Prolong DAPI mounting medium (Invitrogen) and viewed under a fluorescent or confocal microscope.

### mRNAseq analysis

mRNA was isolated from CPC (hCPC1–3), MSC (19, 33, 45) and HDF (F1,F2, F3) as described (Moscoso *et al*., 2013). RNAseq libraries were constructed with the TruSeq RNA Sample Preparation v2 Kit (Illumina). Libraries were sequenced in single-end mode and 75 bp lengths. Fastq files were demultiplexed using the Casava v1.8.2 pipeline. Sequenced reads were aligned to Ensembl transcriptome v65 on hg19 and quantified using RSEM v.1.2.3. Differential expression analyses were performed using voom from edgeR package v3.0.2 (details in Supplementary Methods).

### RT-qPCR

cDNA first strands were synthesized from 1 μg total RNA with the SuperScript III First-Strand Synthesis System (Invitrogen). Genes of interest (see Supplementary Methods) were measured by quantitative RT-PCR in a Mastercycler Ep-Realplex (Eppendorf) platform, using Power SYBR Green reagents (Applied Biosystems). Cycle conditions were 95 °C for 10 min, followed by 40 cycles of 95 °C for 15 s and 60 °C for 1 min. Quantified values were normalized to *GusB* or *GAPDH*.

### Statistics

Assays were performed three times and data were expressed as mean ± SD; black lines show the p-value summary (***<0.002, **<0.02, *<0.05) of CPC *vs*. HDF or MSC (one-way analysis of variance followed by the Bonferroni multiple comparison test).

## Supplementary information

Supplementary Material

Table S1

Table S2

Table S3

Table S4

Table S5

## Data Availability

The mass spectrometry proteomics data, are deposited in Peptide Atlas (http://www.peptideatlas.org/repository/) and are accessible through the PASS00827 accession number. All transcriptomic data derived from this study are deposited in the Gene Expression Omnibus (GEO) database and are accesible through the GSE84070 accession number.
