# Supplementary material for: Definition of a cell surface signature for human cardiac progenitor cells after comprehensive comparative transcriptomic and proteomic characterization
Source: Sci Rep. 2019 Mar 15;9:4647. doi: 10.1038/s41598-019-39571-x (PMC6420620; doi:10.1038/s41598-019-39571-x)
Supplement: Supplementary file 1 — Supplementary Material [file 41598_2019_39571_MOESM1_ESM.pdf]

Definition of a cell surface signature for human cardiac progenitor cells after comprehensive comparative transcriptomic and proteomic characterization

José Luis Torán, Juan Antonio López, Patricia Gomes-Alves, Susana Aguilar, Carlos Torroja, Marco Trevisan-Herraz, Isabel Moscoso, Maria João Sebastião, Margarida Serra, Catarina Brito, Francisco Miguel Cruz, Juan Carlos Sepúlveda, José Luis Abad, Carlos Galán-Arriola, Borja Ibanez, Fernando Martínez, María Eugenia Fernández, Francisco Fernández-Aviles, Itziar Palacios, Luis R-Borlado, Jesús Vázquez, Paula M Alves, Antonio Bernad.

Corresponding author

Antonio Bernad

Department of Immunology and Oncology, Centro Nacional de Biotecnología (CNB-CSIC), Campus Universidad Autónoma de Madrid, 28049 Madrid, Spain [email: abernad@cnb.csic.es].

Running title: Human cardiac progenitor cell proteome

Fig. S1

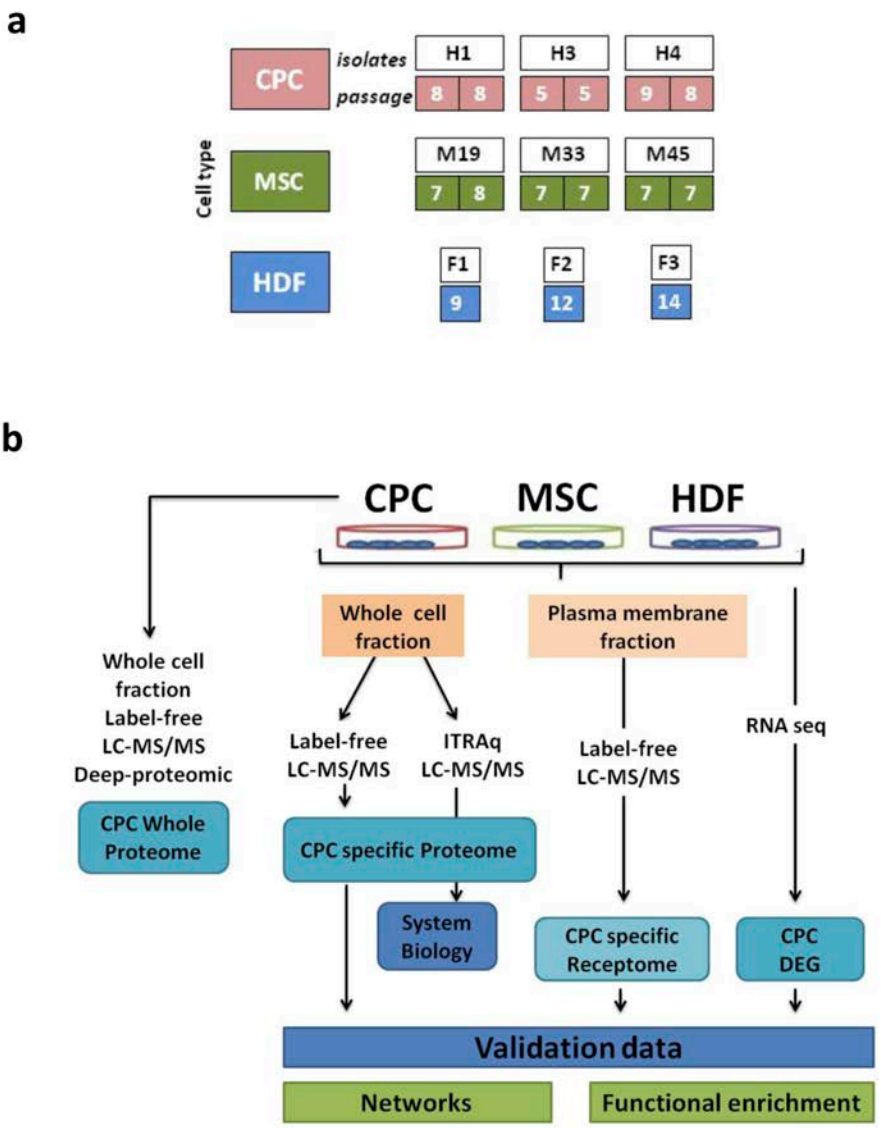

Figure S1. Global scheme of the differential expression analysis. (a) Three independent CPC isolates (CPC1-3; previously referred as H1, H3 and H4, respectively) were compared with three MSC isolates (19, 33, 45) and three HDF isolates (F1-3), at the indicated cell passages. (b) Flow chart of the different analyses carried out with the indicated samples.

Fig. S2

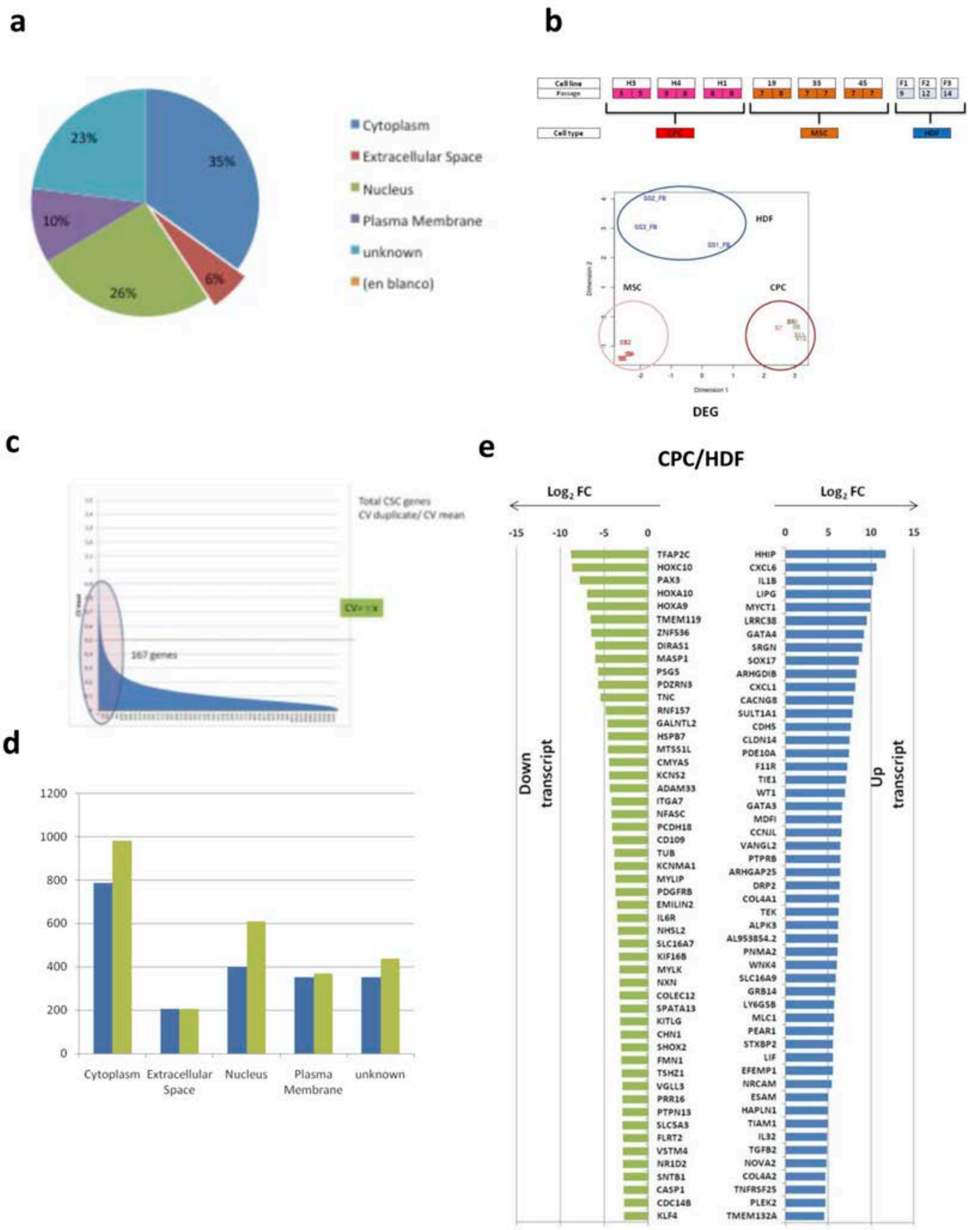

Figure S2. RNAseq analysis of CPC compared with MSC and HDF. (a, b) mRNAseq experiments were carried out and analyzed using the Ilumina platform, with replicates and/or technical duplicates of all samples (see Methods). Analysis of three CPC isolates (CPC1-3) compared with three MSC (19, 33, 45) and three HDF isolates (F1-3) at the indicated passages. CPC data defined 12,242 protein-coding genes with the indicated percentage representation (a). Clustering analysis confirmed that CPC, MSC and HDF cell lineages are quite distant and represent significantly differentiated clusters (b). (c) CV mean values >0.5 (167 out of 11,767 total genes analyzed) were considered as gene expression associates from each passage. (d) Comparative gene distribution among the different cellular subcompartments of MSC (blue bars) compared with CPC (green bars). (e) Plot bar ($\log_2$ FC) of top up- or downregulated genes in CPC (CPC1-3) *vs.* HDF (F1-3).

Fig. S3

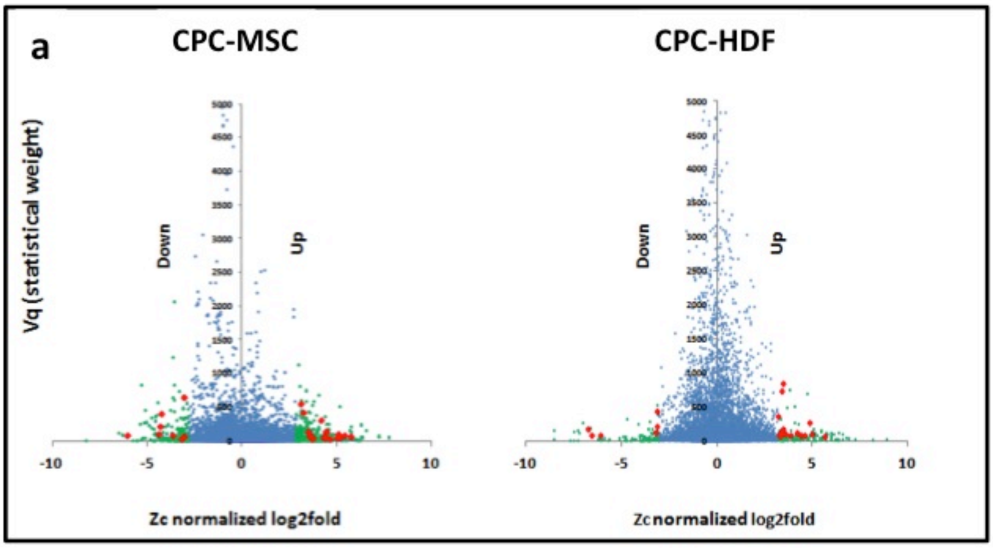

b

CPC vs MSC			
**Protein**	**Description**	**Zq value**	**Peptide number**
IGF2BP3	Insulin-like growth factor 2 mRNA-binding protein 3. GN= IF2B3	5.84	9
IL1B	Interleukin-1 beta	5.41	8
TYB4	Thymosin beta-4. GN=TMSB4X	5.36 (Q)	5
SOD2	Superoxide dismutase [Mn], mitochondrial GN=SODM	3.82	11
COL4A2	Collagen alpha-2(IV) chain. GN=CO4A2	3.77	8
MYH10	Myosin-10	3.45	111
TRPV2	Transient receptor potential cation channel subfamily V member 2	3.20 (Q)	8
IGF2R	Cation-independent mannose-6-phosphate receptor. GN= MPRI	3.19	34
IGFBP7	IBP7_HUMAN Insulin-like growth factor-binding protein 7. GN=IBP7	2.94	12
MYL6B	Myosin light chain 6B. GN=MYL6B	2.92	4
MYH9	Myosin-9. GN=MYH9	2.89	195
LMNB1	Lamin-B1	-2.05	17
LMO7	LIM domain only protein 7	-2.07	59
FAM160B1	Protein FAM160B1. GN=F16B1	-2.35	7
P3H3	Prolyl 3-hydroxylase 3. GN=LEPREL2	-2.47	14
TOM1	Target of Myb protein 1	-2.54	8
LMNA	Prelamin-A/C	-2.65	59
RECQ1	ATP-dependent DNA helicase Q1. GN=RECQL	-2.72	19
PYGB	Glycogen phosphorylase, brain form	-2.91	41
PAI2	Plasminogen activator inhibitor 2. GN=SERPINB2	-3.44	7
GALT1	N-acetylgalactosaminyltransferase 1 GN=GALNT1	-4.11 (Q)	18
H2A1A	Histone H2A type 1-A. GN=HIST1H2AA	-4.55	5

Figure S3. iTRAQ analysis of CPC/HDF and CPC/MSC proteomes. (a) Representative diagrams of up- (+Zq) and downregulated (-Zq) proteins in the CPC vs MSC comparison and CPC vs HDF; Comparison of up- (green) and downregulated (red) proteins, common to the CPC/MSC comparison (left) or CPC/HDF (right). (b) Panel of selected functions down- (green) and up-regulated (red), representative of all cell subcompartments and organized by level of differential expression (Zq), shown as summary of the iTRAQ CPC/MSC comparison; >2 peptides at FDR 0.05%. Q indicates proteins that are similarly upregulated in CPC compared with MSC and HDF.

Fig. S4

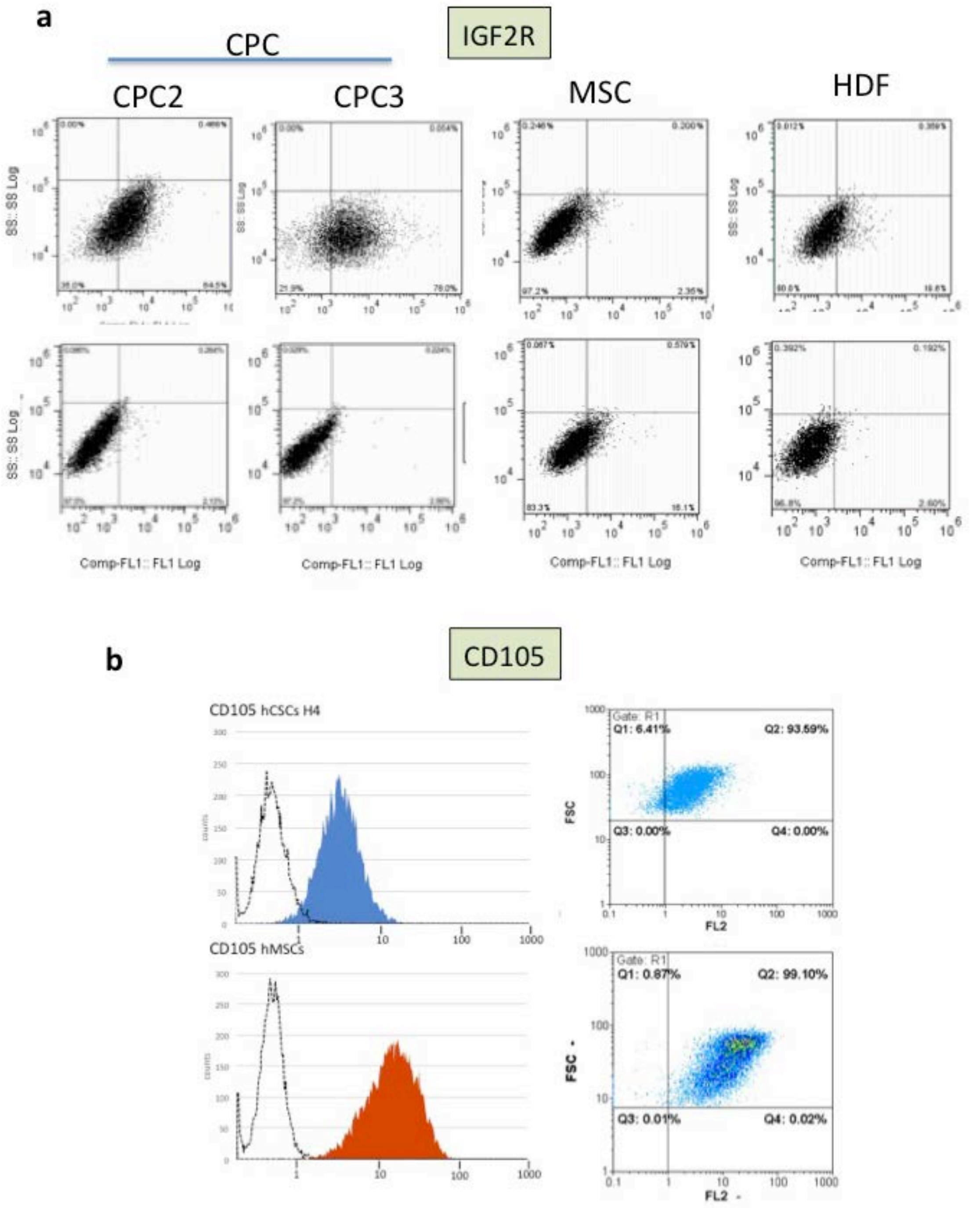

Figure S4. FACS validation of preferentially expressed CPC receptome proteins. (a) FACS analysis of IGF2R expression (upper panels) in CPC (2 and 3) compared MSC (MSC19) and HDF; lower panels show the isotype control stainings. (b) FACS analysis of CD105 expression (blue peak) in CPC-3 compared with MSC (MSC19) (red peak); negative controls with the isotype control stainings are indicated (white peak). The assays were repeated three times; data shown correspond to a representative experiment.

Fig. S5

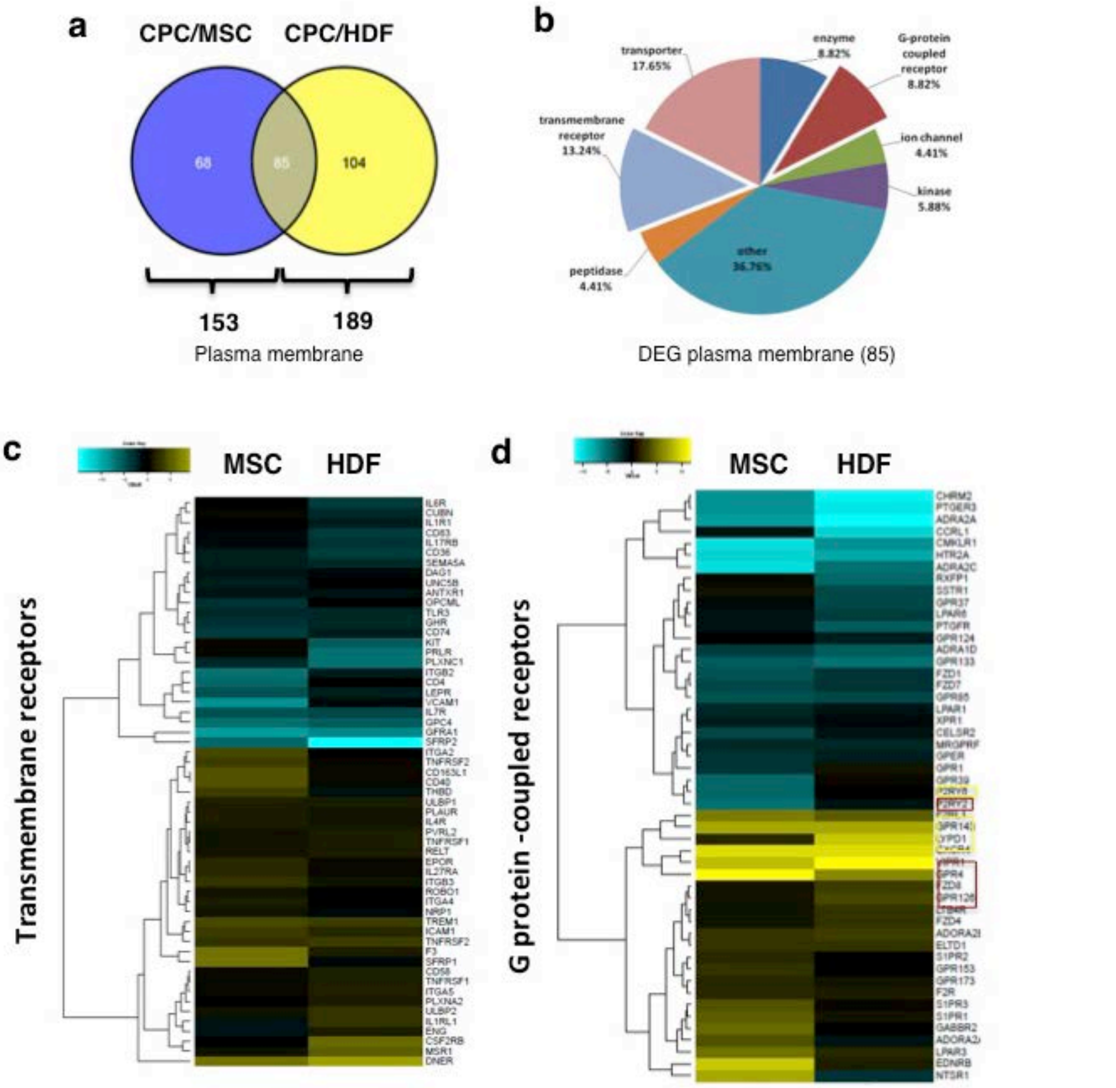

Figure S5. Definition of the minimal core of preferentially expressed plasma membrane proteins in CPC compared with MSC and HDF. (a) Label-free experiments comparing CPC with MSC and HDF; Veen diagram representation of differential upregulated plasma membrane proteins: The specific DEG CPC vs MSC (blue) or DEG CPC *vs.* HDF (yellow) genes and common (grey) are represented; only DEG with p-adjust values <0.02 were considered; 85 proteins were identified exclusively in CPC, with a variety of physiological roles. (b) Relative percentages per specific group of functions, classified by IPA, are indicated. (c,d) Hierarchical clustering of transmembrane receptors (c) and G protein-coupled receptors (d) differentially expressed genes between CPC/MSC and CPC/HDF.

Fig. S6

a

ID symbol	Description
HTR2B	5-hydroxytryptamine (serotonin) receptor 2B, G protein-coupled
ALPP	alkaline phosphatase, placental
ANK3	ankyrin 3, node of Ranvier (ankyrin G)
ATP11C	ATPase, class VI, type 11C
ABCA2	ATP-binding cassette, sub-family A (ABC1), member 2
ABCB5	ATP-binding cassette, sub-family B (MDR/TAP), member 5
BTNL2	butyrophilin-like 2 (MHC class II associated)
CDH13	cadherin 13, H-cadherin (heart)
CELSR1	cadherin, EGF LAG seven-pass G-type receptor 1 (flamingo homolog, Drosophila)
CELSR2	cadherin, EGF LAG seven-pass G-type receptor 2 (flamingo homolog, Drosophila)
CAP2	CAP, adenylate cyclase-associated protein, 2 (yeast)
CEACAM5	carcinoembryonic antigen-related cell adhesion molecule 5
CD274	CD274 molecule
CMKLR1	chemokine-like receptor 1
CGN	cingulin
F3	coagulation factor III (thromboplastin, tissue factor)
COL23A1	collagen, type XXIII, alpha 1
CDCP1	CUB domain containing protein 1
CFTR	cystic fibrosis transmembrane conductance regulator (ATP-binding cassette sub-family C, member 7)
DPP4	dipeptidyl-peptidase 4
DCBLD2	discoidin, CUB and LCCL domain containing 2
DYSF	dysferlin, limb girdle muscular dystrophy 2B (autosomal recessive)
EMB	embigin
EPHA4	EPH receptor A4
EPS15L1	epidermal growth factor receptor pathway substrate 15-like 1
EPB41L1	erythrocyte membrane protein band 4.1-like 1
EPB41L3	erythrocyte membrane protein band 4.1-like 3
EPB41L3	erythrocyte membrane protein band 4.1-like 3
FADS1	fatty acid desaturase 1
FBLIM1	filamin binding LIM protein 1
GRK5	G protein-coupled receptor kinase 5
GPRC5A	G protein-coupled receptor, family C, group 5, member A
GABBR1	gamma-aminobutyric acid (GABA) B receptor, 1
GNAQ	guanine nucleotide binding protein (G protein), q polypeptide
HSPG2	heparan sulfate proteoglycan 2
ITGA3	integrin, alpha 3 (antigen CD49C, alpha 3 subunit of VLA-3 receptor)
ITGA4	integrin, alpha 4 (antigen CD49D, alpha 4 subunit of VLA-4 receptor)
ITGAV	integrin, alpha V
IL1R1	interleukin 1 receptor, type I
IL4R	interleukin 4 receptor
KRT6A	keratin 6A
KRTCAP2	keratinocyte associated protein 2
LRRC7	leucine rich repeat containing 7
LYVE1	lymphatic vessel endothelial hyaluronan receptor 1
MAGT1	magnesium transporter 1
HLA-A	major histocompatibility complex, class I, AB
HLA-A	major histocompatibility complex, class I, A
MPP5	membrane protein, palmitoylated 5 (MAGUK p55 subfamily member 5)
MOK	MOK protein kinase
NKTR	natural killer-tumor recognition sequence
NECAP1	NECAP endocytosis associated 1
NLGN1	neuroligin 1
PDZD2	PDZ domain containing 2
PLIN2	perilipin 2
PLAUR	plasminogen activator, urokinase receptor
PEAR1	platelet endothelial aggregation receptor 1
KCNT1	potassium channel, subfamily T, member 1
KCNN2	potassium intermediate/small conductance calcium-activated channel, subfamily N, member 2
PPFIA4	protein tyrosine phosphatase, receptor type, f polypeptide (PTPRF), interacting protein (liprin), alpha 4
PTPRN2	protein tyrosine phosphatase, receptor type, N polypeptide 2
RIPK2	receptor-interacting serine-threonine kinase 2
ARHGEF26	Rho guanine nucleotide exchange factor (GEF) 26
RUFY3	RUN and FYVE domain containing 3

ID symbol	Description
SEMA7A	semaphorin 7A, GPI membrane anchor (John Milton Hagen blood group)
SLC12A2	solute carrier family 12 (sodium/potassium/chloride transporters), member 2
SLC19A2	solute carrier family 19 (thiamine transporter), member 2
SLC34A1	solute carrier family 34 (sodium phosphate), member 1
SLC39A14	solute carrier family 39 (zinc transporter), member 14
SLC4A3	solute carrier family 4, anion exchanger, member 3
SPTAN1	spectrin, alpha, non-erythrocytic 1
STIM1	stromal interaction molecule 1
SSX2IP	synovial sarcoma, X breakpoint 2 interacting protein
TAS2R3	taste receptor, type 2, member 3
TNS1	tensin 1
TJP2	tight junction protein 2
TJP3	tight junction protein 3
TGFBR2	transforming growth factor, beta receptor II (70/80kDa)
TRPC4	transient receptor potential cation channel, subfamily C, member 4
TNFRSF10C	tumor necrosis factor receptor superfamily, member 10c, decoy without an intracellular domain
TNFRSF10D	tumor necrosis factor receptor superfamily, member 10d, decoy with truncated death domain
TNFRSF19	tumor necrosis factor receptor superfamily, member 19
TUSC3	tumor suppressor candidate 3
USP6NL	USP6 N-terminal like
VMP1	vacuole membrane protein 1
VLDLR	very low density lipoprotein receptor

b

Symbol	CPC/MSC	CPC/ HDF	Gene Name
**EPB41L3**	10.738	2.271	erythrocyte membrane protein band 4.1-like 3
**CDH5**	8.927	7.999	cadherin 5, type 2 (vascular endothelium)
**CD200**	5.648	10.719	CD200 molecule. OX-2
**ARHGEF2**	5.077	3.259	Rho guanine nucleotide exchange factor (GEF) 26
**F11R**	4.635	7.027	F11 receptor
**DYSF**	4,02	6.251	dystrophin related protein 2
**SLC7A1**	3.725	2.129	solute carrier family 7 (amino acid transporter light chain, y+L system), member 1
**ICAM1**	3.557	4.438	intercellular adhesion molecule 1
**JUP**	2,72	2.463	junction plakoglobin
**SEMA4B**	2.129	2.765	sema domain, (semaphorin) 4B
**TNFRSF10D**	1.741	2,12	tumor necrosis factor receptor superfamily, member 10d
**CNTNAP1**	1.729	1.551	contactin associated protein 1
**PPFIA3**	1.541	1.722	protein tyrosine phosphatase, receptor type, F
**TTYH3**	1,31	0,626	tweety homolog 3
**TNFRSF10B**	1.252	1.767	tumor necrosis factor receptor superfamily, member 10b
**EFNB1**	1,06	1,4	ephrin-B1
**CCDC127**	0,566	0,938	family with sequence similarity 127, member A

Figure S6. (a) Complete list of specific CPC membrane proteins; proteins shadowed in yellow were not found in the membrane-proteome approach[4]. (b) List of the 17 membrane proteins more overexpressed in CPC compared with MSC and HDF, and validated both by proteomics and genomics analyses. The list shows the logFC values of the CPC/MSC and CPC/HD RNAseq comparisons ordered by the CPC/MSC differences.

Gene	CDC	CDC	CPC	ckit-CSC	ckit-CSC(Fr)	Sca1-CSC (Fr)	Bmi1-CPC (Fr)
Membrane	m	h	h	h	m	m	m
CD105/ END/ENG	+	+	+ (p,F)	+	+	+(p)	+
CD31/PECAM1	+/-	+	+ (p)	+	+	+(p)	+
CD90/Thy1	+	+	+ (F)	+	+	+	+
CDH5/ VE-Cadherin	+	+	+	+	+	+(p)	+
CD29/ ITGB1	+	+	+ (p,F)	+	+	+	+<
CD200/ OX2G	+	+	+	+	++	+	+>
CD44	+	+	+/-(p)	+	+	+	+
CD9 (Tretrasp 4)	nd	+	+ (p)	+	+	+	+
CLIC4	+	+	+	nd	+	+(p)	+
SCA-1	+	nd	+ (*)	+	+/-	+(p,F)	+
CD49a/ ITGA1	nd	+	+/-	+	+	+(p)	+>
CD166/ ALCAM	nd	+	+	+	+	+	+
CD321/F11R/ F11	nd	+	+	nd	nd	+	+>
CD63	nd	+	+/-(p)	nd	+	+/-	+/-<
CD46	nd	+	+ (p)	nd	nd	+/-	+/->
CD47	nd	+	+/-(p)	nd	nd	+	+<
CD55	nd	+	+ (p)	nd	nd	+	+<
CD34	---	+	+/-	---	+	+	+<
CD40	---	---	+	---	---	nd	+/-
CD133/ PROM1	---	+/-	--- (p)	---	---	+	+>

Figure S7. Comparative analysis of the cell surface specific profile of CPC with those corresponding to human cardiosphere-derived cells (hCDC) and ckit+hCSC. Comparative expression analysis of a significant panel of differentially expressed genes (DEG) of hCPC (salmon) with the rest of populations. Those populations were also compared with murine cardiosphere-derived cells (mCDC), ckit+ mCSC, Sca1+ mCSC and Bmi1-CPC. All the qualitative data included correspond to mRNA expression. Expression comparison also includes some of the final markers proposed (CDH5, CD200 and F11R). Genes are ordered attending to the similarity in expression among all the populations; the gradient in green colors indicates different grade of conservation (dark green, top, denotes the more conserved genes). The gradient in red color denotes genes whose expression is less conserved or with low representation in the study. Key: (+) positive expression; (++) high expression; (+/-) intermediate level of expression; (---) not expressed; (nd) not determined; (> <) indicates a tendency on the value affected; (p) indicates that the specific marker has been also validated by proteomics techniques; (F) indicates that the specific marker has been also validated by FACS. Main references used for the analysis are the following: hCDC[8], hCPC[9], mCDC[10], mckit-CSC[11], mSca1-CSC[12] and BMi1-CPC[13]. All data for murine (m) populations were obtained from freshly purified fractions (Fr), a clear difference with human (h) cells.

Fig. S8

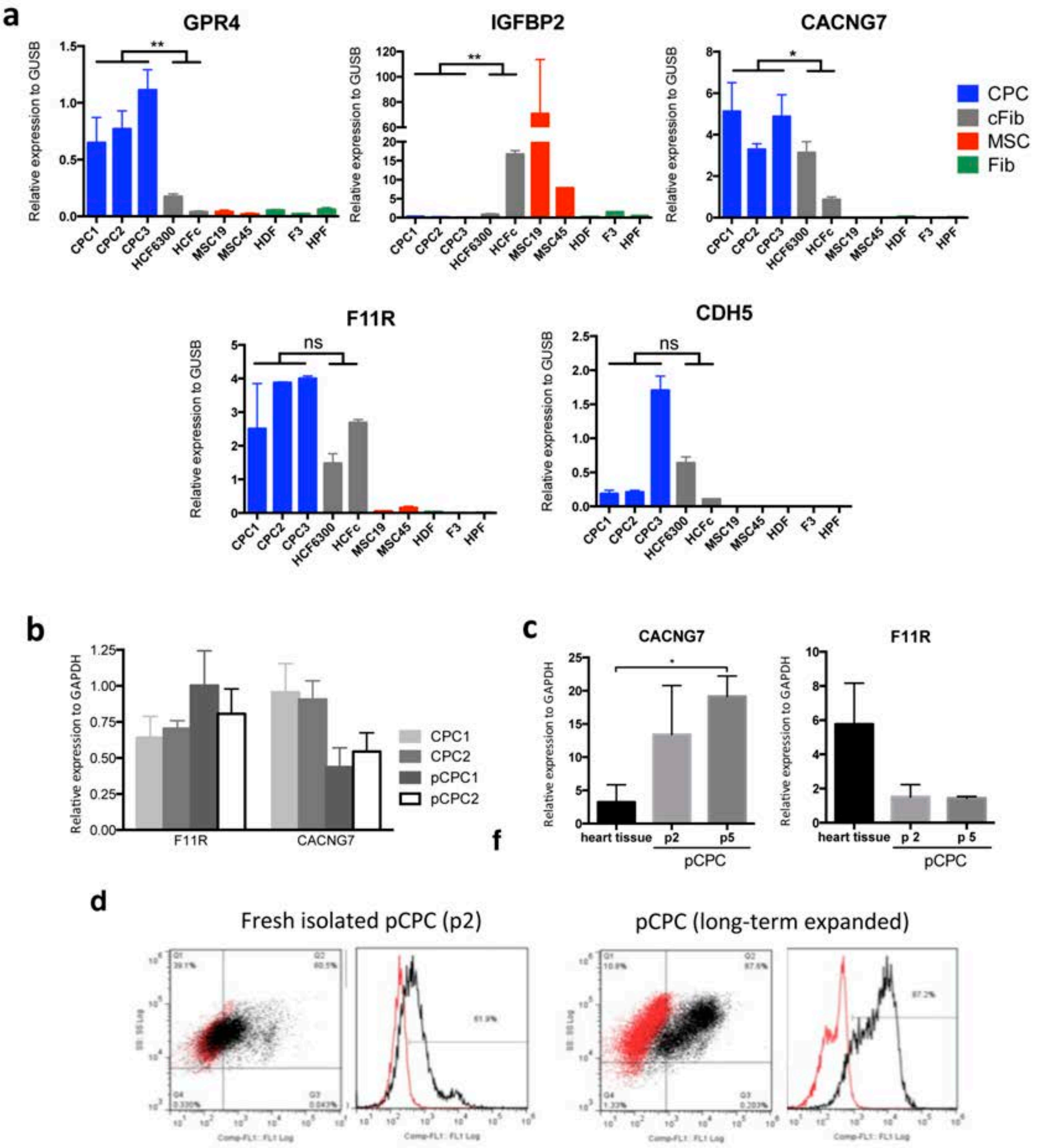

Figure S8. Validation of putative markers of CPC in human and porcine tissue.

(a) RT-qPCR analysis of *GPR4*, *IGFBP2*, *CACNG7*, *F11R* and *CDH5* in human cell samples corresponding to CPC isolates (C1-3; blue bars), cardiac fibroblasts (cFib; grey bars), MSC (red bars) and fibroblasts from different origins (Fib; green bars). The assays were performed three times and data are expressed as mean ± SD; black lines indicate the p-value summary (Mann-Whitney test,***<0.002, **<0.02 *<0.05, ns = not significant) of CPC *vs.* HCF. b) Comparative expression analysis (RT-qPCR) of *F11R* and *CACNG7*, both in long-term expanded human (CPC1 and 2) and porcine (pCPC1 and 2). c) RT-qPCR analysis of *CACNG7 and F11R* expression in porcine samples, both in pCPC and in heart tissue during early isolation stages (p2, p5) The assays were performed three times and data are expressed as mean ± SD; black lines indicate the p-value summary (***<0.002, **<0.02 *<0.05, ns = not significant) of pCPC *vs.* heart tissue (one-way analysis of variance followed by the Bonferroni multiple comparison test). (d) FACS analysis of CDH5 expression in freshly isolated cardiac pig cells (left; p2), compared with long-term expanded pCPC3 (right); CDH5 (black); isotype negative control (red).

Supplementary Tables

Table S1. Complete list of DEG in CPC derived from RNAseq

Table S2. Complete list of proteins identified by label-free proteomics, organized by preferential expression in CPC, MSC and HDF. Organization based on subcellular location.

Table S3. Complete list of CPC/MSC and CPC/HDF ITRAQ comparisons. Cells in green indicate proteins upregulated (+Zq) and cells in red indicate downregulated in CPC (-Zq). Color intensity stratifies expression differences.

Table S4. Complete list of CPC plasma membrane compartment

Table S5. Complete list of DEG CPC plasma membrane compartment

Supplementary experimental procedures

Human and porcine cardiac stem/progenitor cell (CSC/CPC) isolation and culture

CPC samples were cultured in the same growth conditions used for the CAREMI clinical trial (EudraCT 2013-001358-81). Starting material was obtained from the right atria appendage. Tissue samples were minced into small pieces (<1 mm^3) and treated with collagenase type 2 (Worthington Biochemical Corporation, Lakewood, NJ, USA) for 3 cycles of 30 min each to obtain a cellular suspension. Cardiomyocytes were removed by centrifugation and filtration using 40 µm cell strainers. Cardiac stem/progenitor cells were obtained after immunodepletion of CD45-positive cells and immunoselection of CD117 (c-kit)-positive cells, using specific microbeads (MiltenyiBiotech, Bergish Gladbach, Germany) and following manufacturer recommendations. After isolation, cells were seeded in Matrigel (BD Biosciences, Madrid, Spain) coated plates in isolation medium (DMEM/F12 medium supplemented with 10% fetal bovine serum embryonic stem cell qualified (FBS ESCq), L-Glutamine (2 mM), Penicilline- Streptomycine (100 U/mL and 100 µg/mL), bFGF (10ng/mL) and ITS (Invitrogen, Madrid, Spain and Saint-Aubin, France), IGF-II (30ng/mL) and EGF (20ng/mL) (Peprotech, Neuilly-sur-Seine, France) and hEPO (Sigma-Aldrich, Madrid, Spain) and were grown at 37°C in 3% O_2 atmosphere, thereby facilitating proper functioning and mimicking physiologic/pathologic conditions1. One week after cell seeding, growing medium, which is a combination of DMEM/F12 and Neurobasal medium (1:1) supplemented with 10% FBS ESCq, L-Glutamine, Penicilline-Streptomycine, B27 (1X), N2 (1X), β-mercaptoethanol (50µM), ITS and growth factors (bFGF, IGF-II, EGF) replaced the isolation medium and cells were thereafter grown in this medium at 3% O2 atmosphere for some passages. Porcine heart samples were processed essentially as previously described for human samples. CD45-c-kit+ fraction was cultured in collagen-coated plates at 37°C in 3% O_2 atmosphere, in growing medium and analyzed in passages 2 and 5.

Human bone marrow-derived MSC and human fibroblasts

Human bone marrow-derived MSC (hMSC) and human dermal fibroblasts (HDF) were obtained from the Inbiobank Stem Cell Bank (www.inbiobank.org). Briefly, cadaver bone marrow was harvested from brain-dead donors under the supervision of the Spanish National Transplant Organization (*Organización Nacional de Trasplantes*, ONT). Relatives gave informed consent. Each sample donor was tested and found negative for: HIV-1/2, hepatitis B-C, cytomegalovirus and mycoplasma. All cells were processed at Inbiobank following manufacturing procedures based on ISO9001:2000 in GMP conditions. 05+ Phenotypes were described previously[3]. The hMSC displayed a typical CD29+, CD73+ (SH3 and SH4),

CD1 (SH2), CD166+, CD34-, CD45- and CD31- phenotype. In the presence of specific differentiation factors, these cells were able to differentiate into osteocytes, chondrocytes and fatty cell lineages.

Other fibroblastic cell lines used in the study were as follow: HCF (ScienceCell, cat. Number 6300), HCF-c (PromoCell, cat. Number C-12375) and HPF-c (PromoCell, cat. Number C-12360). MSC and all fibroblastic cell lines were cultured in optimal conditions for each, in low-glucose DMEM (Sigma-Aldrich) with 10% FBS.

Immunohistochemical analyses

For immunohistochemistry, heart samples were fixed in 4% paraformaldehyde (PFA) (overnight, 4ºC) and cryopreserved in 30% saccharose, frozen in OCT compound, and sectioned in 8-mm sections on a cryostat. Heart immunohistochemistry and immunocytochemistry (ICC) have been described in detail (Valiente-Alandí et al., 2015). After blocking with 5% BSA (bovine serum albumin)/PBS (overnight (O/N), 4ºC) slides were then incubated in 1% BSA/PBS with the indicated primary antibodies (1-2 h, 37°C) (anti-CDH5, antiF11R and anti-CD200; Table Supp Materials and Methods). Slides were washed three times in PBS/1% BSA and incubated in PBS/1% BSA with appropriate secondary antibodies (1 h, 37°C). Images were captured with a Zeiss LSM 700 or Leica TCS SP5.

Proteomics analysis

Cells from CPC isolates H1, H3 or H4 were cultured; after repeated washing in PBS, cell pellets (5-8 x 10^7) were collected and aliquoted. For protein extract preparation, cell pellets were resuspended in lysis buffer (50 mM Tris-HCl pH8.5, 4% SDS, 50 mM DTT), boiled (5 min) and incubated (30 min, room temperature) for full protein solubilization. Total protein (~200 mg) was digested using a filter-aided sample preparation protocol (FASP, Protein Digestion Kit, Expedeon) following manufacturer's instructions. Protein extracts were diluted in buffer UA (8 M urea in 0.1 M Tris-HCl pH 8.5) and loaded onto 30K centrifugal filter devices. Denaturation buffer was replaced by washing with buffer UA. Proteins were alkylated using 50 mM iodoacetamide in buffer UA (20 min in the dark) and excess alkylation reagent was eliminated by three washes with buffer UA and three washes with 50 mM ammonium bicarbonate. Proteins were digested (37ºC, overnight) with modified trypsin (Promega) in 50 mM ammonium bicarbonate at a 40:1 protein: trypsin (w/w) ratio. The resulting peptides were eluted by centrifugation with 50 mM ammonium bicarbonate (twice) and 0.5 M sodium chloride. TFA was added to a final concentration of 1%, the peptides were desalted on C18 Oasis-HLB cartridges (Waters) and dried for further analysis.
The resulting tryptic peptides were dissolved in 0.1% formic acid, loaded onto the nLC-MS/MS system for on-line desalting on C18 cartridges, and analyzed by nLC-MS/MS using a C-18 reverse-phase nanocolumn (75 mm ID x 50 cm, 3 mm particle size, Acclaim PepMap 100 C18, Thermo-Fisher) in a continuous acetonitrile gradient consisting of 0-30% B in 180 min, 30-43% in 5 min and 43-90% B in 1 min (A= 0.5% formic acid; B=90% acetonitrile, 0.5% formic acid). Peptides were eluted at a flow rate of ~200 nL/min from the reverse phase nano-column to an emitter nanospray needle for real time ionization and peptide fragmentation on orbital ion trap mass spectrometers (Orbitrap Elite mass spectrometer, Thermo-Fisher). To increase proteome coverage, tryptic peptides were fractionated by cation exchange chromatography (Oasis HLB-MCX columns), desalted and analyzed as above.
The enriched membrane fraction from CPC (H4 isolate, Coretherapix), MSC and HDF, using the optimized extraction protocol[4]. was processed by off-line fractionation (medium cation exchange chromatography, MCX) prior to nLC-MS/MS analysis. Six MCX fractions were analyzed by nLC-MS/MS in the Orbitrap XL equipment, using a 3-h gradient. Approximately 20% of the proteins identified were assigned as plasma membrane proteins, including several receptors and proteins with numerous predicted transmembrane domains (TMD).

Database search

To identify peptides, MS/MS spectra were searched with the SEQUEST HT algorithm implemented in Proteome Discoverer 1.4.0.29 (Thermo Scientific). For database searching at the Uniprot database (which contains all human sequences; March 06, 2013; 70024 entries; Mann's lab contaminants), search

parameters were: trypsin digestion with 2 maximum missed cleavage sites, precursor and fragment mass tolerances of 800 ppm and 1.2 Da, respectively for Elite files, or 2 Da and 0.02 Da, respectively for QExactive files, carbamidomethyl cysteine as fixed modification and methionine oxidation as dynamic modifications.

For iTRAQ-labeled peptides, N-terminal and Lys iTRAQ modifications were selected as a fixed modification. Results were analyzed using the probability ratio method with additional filtering for a precursor mass tolerance of 15 ppm; a false discovery rate (FDR) for peptide identification was calculated based on the search results against a decoy database using the refined method. The iTRAQ reporter ion intensities were retrieved from MS/MS scans by QuiXoT software and used as inputs to the weighted spectrum, peptide and protein statistical (WSPP) model to obtain peptide and protein abundance changes. Using the ontologies and annotations included in the GO database, WSSP was used to assess statistically significant changes at the protein function level.

Peptide quantification and statistical analysis

Peptides were quantified using QuiXoT quantitative proteomics software (Jorge et al., 2009). As a starting point, $X_{qps} = \log_2(A/B)$ $X_{???} = \log_{2(A/B)}$ was calculated, where *A* and *B* are the intensity of reporter ions being compared, retrieved in the MS/MS scans. These ratios were corrected based on the distribution of masses of the iTRAQ reporter ions.

Statistical analyses were based on the WSPP statistical model[5] a random effects model that considers five sources of variance: spectrum-fitting, scan, peptide, protein and functional category levels. A weight W_{qps} was associated to each spectrum, using the maximum intensity of each pair of iTRAQ reporter ions compared. The overall $\log_2$ ratio of each peptide, X_{qp}, was calculated as a weighted average of the scans matching each peptide. The $\log_2$ ratio of each protein, X_q, was similarly calculated, using the weighted average of all the peptides that identify the protein studied. The final X_q value was corrected by subtracting the grand mean of each experiment. The weights for spectra, W_{qps}, were corrected based on the spectrum level variance, σ_S^2. The weight for peptides, W_{qp}, was then calculated by adding the weights of all spectra matching that peptide and considering variance, σ_P^2, and the weight for the protein, W_q, was calculated by adding the weights of all peptides associated to each protein, considering protein variance, σ_Q^2. Standardized variables, Z_{qps}, Z_{qp} and Z_q, were defined at each level as the mean-corrected $\log_2$ ratio, expressed in units of the corresponding standard deviation. Further details can be found in previous reports[5-7].

Bioinformatics identification

For peptide identification, all spectra were analyzed with Proteome Discoverer (version 1.4.0.29, Thermo Fisher Scientific), using a Uniprot database containing all human and chicken protein sequences (November 23, 2011). For searching, parameters were selected as follows: trypsin digestion with 2 maximum missed cleavage sites, precursor and fragment mass tolerances for Elite of 600 ppm and 1200 mmu respectively (2 Da and 0.02 Da respectively for QExactive), carbamidomethyl cysteine as fixed modification, and methionine oxidation as dynamic modifications. Peptide identification was validated using the probability ratio method and FDR were calculated using inverted databases and the refined method.

RNA-Seq analysis

Sequenced reads were quality-controlled and pre-processed using cutadapt v1.6 to remove adaptor contaminants. Resulting reads were aligned and gene expression quantified using RSEM v1.1.19, over human reference GRCh37 and Ensembl gene build 65. Only genes with at least 1 count per million in at least three samples were considered for statistical analysis. Data were then normalized and differential expression tested using the bioconductor package EdgeR v3.0.8. We considered as differentially expressed those genes with a Benjamini-Hochberg adjusted p-value ≤0.05. For the set of differentially expressed genes, functional analysis was performed using topGO v2.10 Bioconductor R package, with

annotations from org.Hs.eg.db and GO.db v 2.8. For the functional analysis, genes were classified by subcellular compartment (nucleus, cytoplasm, plasma membrane, extracellular) according to GO annotations. Enrichment was performed using the full list of equally localized genes as reference. Top biological processes and molecular functions were selected using the Weighted Fisher method implemented by topGO with P<0.01.

Table. List of antibodies used in the study

Primary anti-human antibodies	*Reference*	*Commercial provider*
Anti-CD200 antibody	AF2724	R&D Systems, Minneapolis, MN
Anti-IGF-II R antibody	AF2447	R&D Systems, Minneapolis, MN
Anti-JAM-A antibody	MAB1103	R&D Systems, Minneapolis, MN
Anti-VE-Cadherin/CD144 antibody	MA5-17050	Thermo Scientific, Waltham, MA
Anti-CD130 Monoclonal antibody	PA5-28932	Thermo Scientific, Waltham, MA
Anti-CD59 Monoclonal antibody	MA5-17046	Thermo Scientific, Waltham, MA
Anti-CD105 Monoclonal Antibody	MA4-17041	Novus Biologicals, UK
Anti-CD26 Monoclonal Antibody	MA5-13562	Thermo Scientific, Waltham, MA
Anti-CACNG7	Ab110054	Abcam, Cambridge, MA
Anti-CD9 Antibody	Ab92726	Abcam, Cambridge, MA
Anti-GPCR GPR4 Antibody	Ab75330	Abcam, Cambridge, MA
Anti-α-tubulin	CP06	Calbiochem, La Jolla, CA, USA

Secondary antibodies	*Reference*	*Commertial provider*
Goat Anti-rabbit HRP	P0448	Dako, Glostrup Denmark
Rabbit Anti-Mouse-HRP	P0260	Dako, Glostrup Denmark
Rabbit Anti-Goat HRP	P0449	Dako, Glostrup Denmark
Rabbit Anti-Goat IgG (H+L)-FITC	6160-02	Southern Biotech, Birminghan, AL, USA
Goat anti-Rabbit IgG (H+L) Secondary Antibody, Alexa Fluor® 546 conjugate	A-11035	Invitrogen, Carlsbad, CA, USA
Goat anti-Mouse IgG (H+L) Secondary Antibody, Alexa Fluor® 546 conjugate	A-11030	Invitrogen, Carlsbad, CA, USA

Table. List of RT-qPCR primers used in the study

Primer	Sequence (5´-3´)
GPR4 Forward	TAATGCTAGCGGCAACCACACGTGGGAG
GPR4 Reverse	TCCAGTTGTCGTGGTGCAG
SERCA Forward	GAGAACGCGCACACCAAGA
SERCA Reverse	TTGGAGCCCCATCTCTCCTT
CDH5 Forward	TCACCTTCTGCGAGGATATGG
CDH5 Reverse	GAGTTGAGC ACCGACACATC
F11R Forward	TCGAGAGGAAACTGTTGTGC
F11R Reverse	GAAGAAAAGCCCGAGTAGGC
F11R Forward (porcine)	TCTTGTGCTCCCTGACGTTG
F11R Reverse (porcine)	AATTTCCACTCCACACGGGG
CACNG7 Forward (h & p)	TAAAGAACCAAGCCCACCAC
CACNG7 Reverse (h & p)	TCAGCCTCTTCCTCGTGTTC
IGFBP2 Forward	GCCCTCTGGAGCACCTCTACT
IGFBP2 Reverse	GCCCTCTGGAGCACCTCTACT
CD9 Forward	GAGGCACCAAGTGCATCAA
CD9 Reverse	AGCCATAGTCCAATGGCAAG
ECE1 Forward	GAAGCGGCTGGTGGTGTTGGTG
ECE1 Reverse	GGTTGGCCTTGATCCAGC
DAB2IP Forward	TGGACGATGTGCTCTATGCC

DAB2IP Reverse	GGATGGTGATGGTTTGGTAG
ITGA5 Forward	AAGAGCCG GATAGAGGACAAG
ITGA5 Reverse	AAGTGAGGTTCAGGGCATTC
GusB Forward	CAACGAGCCTGCGTCCCACC
GusB Reverse	ACGGAGCCCCCTTGTCTGCT
GAPDH Forward	AACTGCTTGGCACCCCTGGC
GAPDH Reverse	CTGGAGAGCCCCTCGGCCAT

Supplementary references

1. Lauden, L., *et al*. Allogenicity of human cardiac stem/progenitor cells orchestrated by programmed death ligand 1. *Circ Res.* **112**, 451-464 (2013).
2. Boukouaci W., *et al*. Natural killer cell crosstalk with allogeneic human cardiac-derived stem/progenitor cells controls persistence. Cardiovasc Res. **104**, 290-302 (2014).
3. Carcamo-Orive I., *et al*. Regulation of human bone marrow stromal cell proliferation and differentiation capacity by glucocorticoid receptor and AP-1 crosstalk. *J Bone Miner Res*. **25**, 2115-25 (2010).
4. Gomes-Alves, P. *et al*. Exploring analytical proteomics platforms toward the definition of human cardiac stem cells receptome. *Proteomics* **15**,1332-1337 (2015).
5. Navarro P., *et al*. General statistical framework for quantitative proteomics by stable isotope labeling, J proteome res **13**, 1234-1247 (2014).
6. Jorge I., *et al*. Statistical Model to Analyze Quantitative Proteomics Data Obtained by 18O/16O Labeling and Linear Ion Trap Mass Spectrometry, Mol Cell Proteomics **8**, 1130-1149 (2009).
7. Jorge I., *et al*. The human HDL proteome displays high inter-individual variability and is altered dynamically in response to angioplasty-induced atheroma plaque ruptura, J Proteomics **106**, 61-73 (2014).
8. hCDC patent (PCT/US2013/043772.CAPRICOR).
9. hCPC patent (United States Patent Application 20160030485.Cortherapix SLU).
10. Machida M., Takagaki Y., Matsuoka R., Kawaguchi N. Proteomic comparison of spherical aggregates and adherent cells of cardiac stem cells. Int J Cardiol. **153**, 296-305 (2011).
11. Sandstedt J., *et al*. Human C-kit+CD45- cardiac stem cells are heterogeneous and display both cardiac and endothelial commitment by single-cell qPCR analysis. Biochem Biophys Res Commun. **443**, 234-448 (2014).
12. Samal R., *et al*. OMICS-based exploration of the molecular phenotype of resident cardiac progenitor cells from adult murine heart. J. of proteomics **75,** 5304–5315 (2012).
13. Valiente-Alandi I., Albo-Castellanos C., Herrero D., Sanchez I. and Bernad, A. Bmi1^{+} cardiac progenitor cells contribute to myocardial repair following acute injury. Stem Cell Res Ther. **7**, 100 (2016).
